# The PHEM-B toolbox of methods for incorporating the influences on Behaviour into Public Health Economic Models

**DOI:** 10.1186/s12889-024-20225-1

**Published:** 2024-10-12

**Authors:** Hazel Squires, Michael P. Kelly, Nigel Gilbert, Falko Sniehotta, Robin C. Purshouse, Leandro Garcia, Penny Breeze, Alan Brennan, Benjamin Gardner, Sophie Bright, Alastair Fischer, Alison Heppenstall, Joanna Davan Wetton, Monica Hernandez-Alava, Jennifer Boyd, Charlotte Buckley, Ivo Vlaev, Robert Smith, Ali Abbas, Roger Gibb, Madeleine Henney, Esther Moore, Angel M. Chater

**Affiliations:** 1 University of Sheffield, Sheffield, UK; 2 University of Cambridge, Cambridge, UK; 3 University of Surrey, Surrey, UK; 4 Heidelberg University, Heidelberg, Germany; 5 Queen’s University Belfast, Belfast, UK; 6 London School of Hygiene and Tropical Medicine, London, UK; 7 University of Glasgow, Glasgow, UK; 8 University College London, London, UK; 9 University of Stirling, Stirling, UK; 10 University of Warwick, Warwick, UK; 11 Dark Peak Analytics, Sheffield, UK; 12 Cambridge, UK

**Keywords:** Public health, Behaviour, Theory, Simulation, Economic, Sociology, Psychology, Complexity, Maintenance, Methods

## Abstract

**Background:**

It is challenging to predict long-term outcomes of interventions without understanding how they work. Health economic models of public health interventions often do not incorporate the many determinants of individual and population behaviours that influence long term effectiveness. The aim of this paper is to draw on psychology, sociology, behavioural economics, complexity science and health economics to: (a) develop a toolbox of methods for incorporating the influences on behaviour into public health economic models (PHEM-B); and (b) set out a research agenda for health economic modellers and behavioural/ social scientists to further advance methods to better inform public health policy decisions.

**Methods:**

A core multidisciplinary group developed a preliminary toolbox from a published review of the literature and tested this conceptually using a case study of a diabetes prevention simulation. The core group was augmented by a much wider group that covered a broader range of multidisciplinary expertise. We used a consensus method to gain agreement of the PHEM-B toolbox. This included a one-day workshop and subsequent reviews of the toolbox.

**Results:**

The PHEM-B toolbox sets out 12 methods which can be used in different combinations to incorporate influences on behaviours into public health economic models: collaborations between modellers and behavioural scientists, literature reviewing, application of the Behaviour Change Intervention Ontology, systems mapping, agent-based modelling, differential equation modelling, social network analysis, geographical information systems, discrete event simulation, theory-informed statistical and econometric analyses, expert elicitation, and qualitative research/process tracing. For each method, we provide a description with key references, an expert consensus on the circumstances when they could be used, and the resources required.

**Conclusions:**

This is the first attempt to rigorously and coherently propose methods to incorporate the influences on behaviour into health economic models of public health interventions. It may not always be feasible or necessary to model the influences on behaviour explicitly, but it is essential to develop an understanding of the key influences. Changing behaviour and maintaining that behaviour change could have different influences; thus, there could be benefits in modelling these separately. Future research is needed to develop, collaboratively with behavioural scientists, a suite of more robust health economic models of health-related behaviours, reported transparently, including coding, which would allow model reuse and adaptation.

**Supplementary Information:**

The online version contains supplementary material available at 10.1186/s12889-024-20225-1.

## Background

### Public health interventions and health economic modelling

Public health interventions may include actions or activities that aim to make a person or population change behaviours to improve their health. They are often multi-component and operate within complex systems, which means that there is not a clear boundary around the system and the sum of individual intervention effects is not equal to the outcomes at the population level due to the interactions between heterogeneous individuals and the influence of their environment. Thus, effects of public health interventions may be non-linear, and sometimes unexpected, at the macro level, with the wider context impacting intervention effectiveness [1].

Health economic models are used to predict the difference in costs and effectiveness between current practice and alternative interventions, usually over an individual’s lifetime, to capture all impacts of the interventions to inform policy decisions about how best to spend limited resources [2, 3]. The benefit of these models is that they can synthesise evidence from a range of sources and simulate possible future costs and outcomes of alternative interventions. Existing health economic models do not typically incorporate the determinants of individual and population behaviours that influence long term effectiveness, yet it is essential to understand how public health interventions work in order to attempt to predict long-term outcomes of interventions.

Health economic models include simple arithmetic calculations, cohort state transition models, and individual level simulations [4]. More flexible individual-level health economic models can be useful when decision makers want to understand the different impacts of interventions upon individuals or different groups of the population to reduce health inequalities [5] or when outcomes depend on time or event-dependent interactions [6]. Typically, within these models, a population is synthesised to match the characteristics of the real population of interest, so that every individual has their own attributes (e.g. age, socioeconomic status, Body Mass Index (BMI)), which can be updated over time [7]. These models can then be used to estimate the incidence and progression of diseases and health conditions using epidemiological risk equations, to estimate mortality, to assign different resource use, costs and utilities, to test the impact of alternative interventions, and to report outcomes by relevant subgroups.

### Why is it useful to understand the influences on behaviour to predict behaviour and long-term outcomes of public health interventions?

The effectiveness of public health interventions is dependent upon human behaviour. Behaviour is complex and multifaceted, and shaped by many influences which change over time. In some health economic models of public health interventions, only biological risk factors for disease, such as BMI, are incorporated [8], without including the contributing behaviours such as eating and physical activity. In others, behavioural risk factors (e.g., smoking) are included, but the influences upon these behaviours, such as those related to capability (e.g. knowledge/behavioural regulation), opportunity (e.g. environmental context, social influences) and motivation (e.g. beliefs, emotion, reinforcement), are not explicitly considered [9]. The impact of the intervention is generally estimated based on a single study or a meta-analysis of the effectiveness of an intervention [3] and effectiveness evidence is often limited to 6 – 12 months follow up [10–12]. There is a dearth of evidence about behavioural maintenance resulting from interventions and there are no standard approaches for estimating the impacts of the intervention beyond the end of study follow up. Assumptions range from maintaining the effectiveness over an individual’s lifetime, to reverting to the outcomes of the comparator either immediately or gradually over some time period [13, 14]. These assumptions are usually based on little theory or evidence, and generally do not vary by individual characteristics or intervention type.

**Fig. 1:**
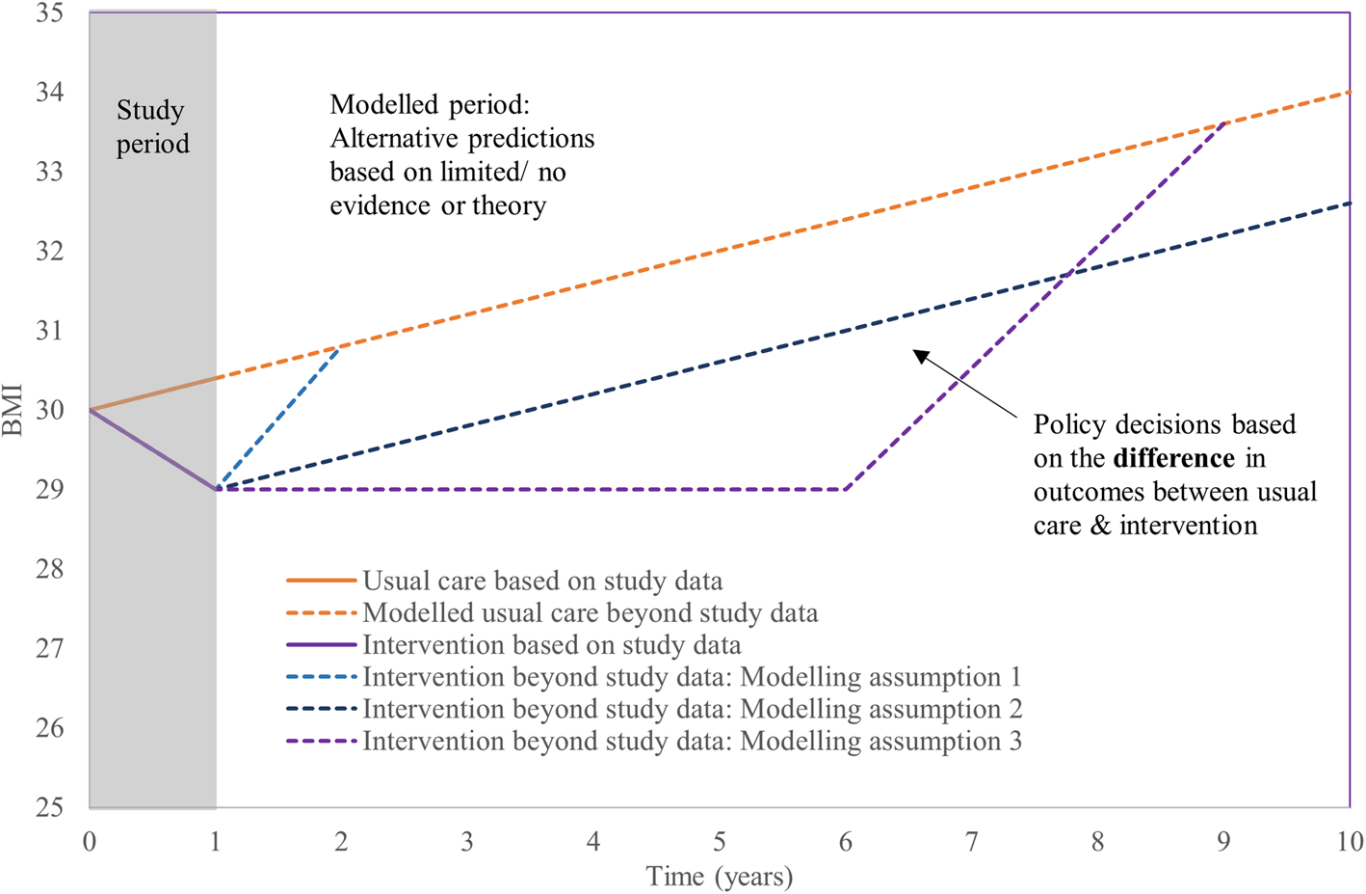
Illustration of the importance of assumptions beyond study follow up legend: The three blue/ purple dashed lines each represent an alternative modelling assumption about the effectiveness of the intervention beyond the study period: Body Mass Index (BMI) returning to usual-care levels within 1 year; BMI slowly increasing post-intervention, at the same rate as those under usual care; or BMI remaining at the lower post-intervention level for 5 years and then returning to what would be predicted under the usual care scenario over a further 3 years

Figure 1 shows some potential alternative modelling assumptions beyond study follow up for a hypothetical intervention which reduces BMI (either by increasing physical activity or improving diet). The cost-effectiveness results are based on the average differences between usual care and the interventions, which may be dramatically different depending on which assumptions are chosen [13]. For simplicity, this hypothetical example shows only usual care and one intervention, with three alternative modelling assumptions for the cohort. However, there are often multiple interventions to compare, and it may not be appropriate to make the same extrapolation assumptions for each intervention or each individual, given that there are many factors that will affect behaviours over time.

During model development, assumptions about the effectiveness of the interventions are often treated as an ‘add on’ to the main modelling of the current system. This may be because health economic modelling traditions were developed for the evaluation of clinical interventions, where effectiveness evidence tends to come from randomised controlled trials and the reasons *why* an intervention works or does not work will not affect policy decisions. Yet, for public health economic evaluation, the benefits of developing a more detailed individual-level simulation model are likely thwarted by the basic assumptions about the intervention effectiveness over the long-term. It is not advisable to attempt to predict future outcomes without any understanding of the mechanism of action – i.e., the processes through which an intervention affects behaviour, such as by increasing motivation [15]. This is particularly important when policy makers are comparing the cost-effectiveness of several alternative interventions which are made up of different behaviour change techniques, some of which are more likely to lead to maintenance of a new behaviour than others [16].

In addition, interventions may affect different behaviours (e.g., eating or physical activity) or different elements of a behaviour domain (e.g., eating fruit and vegetables or salt intake). Intervention strategies may include taxation policies, environmental changes, service provision, or education. Each of these intervention strategies has different mechanisms of action, with some depending more upon changing individual factors, and others on changing the external environment. If the same extrapolation assumptions are made for all interventions this could lead to inappropriate comparisons between the interventions. Moreover, different individuals may be more likely to maintain a behaviour than others, according to some environmental influences, biological factors or psychological attributes, and subsequent inequalities are likely to be important to decision makers. Thus, the underlying evidence, the choice of assumptions, and appropriate representation of uncertainty related to these aspects, is fundamental for informing policy decisions.

### Behavioural theories and frameworks

Research undertaken within other disciplines including psychology, sociology, complexity science and behavioural economics can help to inform assumptions about behaviour beyond intervention study follow up. Many theories and frameworks have been developed to explain human behaviour. These link a set of biological, psychological, social and/or environmental factors to behaviour, offering a bio-psycho-social understanding of behaviour. Such factors can be thought of as potential intervention targets for change and mechanisms of action. The effectiveness of interventions for changing and maintaining behaviour will depend on which influences are targeted, and to what extent changes in these influences (e.g., knowledge), and the behaviour change techniques (e.g., providing information) and intervention strategies (e.g., education) used impact on behaviour. However, there is no one accepted behavioural theory, and there are multiple mechanisms of action and behaviour change techniques [17]. Michie [18] collated 83 behavioural theories which could inform the development of behavioural interventions. Within this review, the three theories for which the most published papers (more than 50%) were identified were: (i) the Transtheoretical Model of Behaviour Change (which includes progression and feedback loops through the stages precontemplation, contemplation, preparation, action and maintenance, allowing for relapse) [19]; (ii) The Theory of Planned Behaviour (which links attitude, subjective norms, perceived behavioural control and intentions to behaviour) [20]; and (iii) Social Cognitive Theory (which links the interaction between the individual, environment and behaviour with behavioural capability, observational learning, reinforcement, expectations and self-efficacy) [21].

Recent literature encourages the use of behavioural theories to inform public health intervention development to understand what works for whom in which contexts [22]. However, many existing studies do not report their theoretical basis [23] and a narrative synthesis of nine systematic reviews found no difference in the effectiveness of interventions that were theory-based versus non-theory-based [24]. The study authors suggested that this could be due to limitations with the theories used, (with two of the most widely used having had calls to be retired [25, 26]) or issues with fidelity and the way in which they were applied.

The Behaviour Change Wheel developed by Michie et al. [27] has become an important framework for developing interventions, because it provides a coherent and comprehensive approach. It was developed based upon a systematic review of the literature and subsequently tested for reliability. The Behaviour Change Wheel includes influences on behaviour at the hub, with a wide range of intervention types and policy options set out in the middle and outer layers of the wheel. There are then tables to facilitate a systematic selection of intervention strategies and behaviour change techniques according to a behavioural analysis. The influences on behaviour at the hub are conceptualised through the COM-B model of behaviour, where Behaviour can be explained by a combination of Capability, Opportunity and Motivation. Capability includes physical (e.g., skills/ strength) and psychological (e.g., knowledge, memory, attention and decision making, behavioural regulation) factors; Motivation includes reflective (e.g. beliefs, intentions, identity) and automatic (e.g. emotion, reinforcement) factors; and Opportunity includes physical (e.g. environmental context and resources) and social (e.g. norms, culture) influences.

Any theory that is used to develop the interventions is generally ignored when they are evaluated within a health economic model. This is partly because health economic modellers tend to use methods developed for the evaluation of clinical interventions, and partly due to limitations of existing behavioural theories, including data limitations to support them and the fidelity with which they are implemented in the interventions. To predict the long-term effectiveness of interventions, it is important to understand the precise content and context of the interventions [1]. Combining behavioural theory with modelling, as has been done in other fields such as natural resource management [28], could help to understand the longer-term impacts of a range of interventions with different mechanisms of action upon individuals with different attributes.

### Why might it be important to consider the influence of social networks and the broader environment?

While many of these behavioural theories focus primarily on individual psychology, there are a range of theories which suggest that behaviour is influenced by others, including our perception of others, and/ or the broader environment, which are discussed in this section.

There is evidence that people do not consider all possible outcomes systematically when making many behavioural decisions (as is assumed in standard economic theory of rational choice). Instead, to cope with complex choices, people use heuristics which are strategies that enable faster decision making whilst only using some information [29, 30]. These heuristics have been shown to lead to predictable patterns of behaviour. Theory associated with ‘nudging’ [31] – that is, ‘any aspect of the choice architecture that alters people’s behaviour in a predictable way without forbidding any options or significantly changing their economic incentives’ – has been extensively used to develop behaviour change interventions [32]. Nudge theory focuses on automatic mechanisms (non-reflective decision making) and how the context can affect these in positive ways, although it does not exclude reflective mechanisms (deliberate, highly cognitive decision making). The mnemonic MINDSPACE (Messenger, Incentives, Norms, Defaults, Salience, Priming, Affect, Commitment and Ego) has been used to set out the most powerful contextual influences on automatic behaviours [32].

Social structure is about the patterns of social relationships within a population. An individual’s behaviour will impact upon these social relationships, and at the same time their social connections will affect their behaviour [33]. For example, a physically active person may join a running club and make friends with other runners, which might increase the amount the person runs. It has been suggested that friends and family influence weight-related behaviours and body weight [34]. People may influence each other through physical or online networks; for instance, social media is often used as a platform to influence behaviour [35, 36]. The interaction of many individuals within a social structure can alter that structure and change social norms. The theory associated with social structure [33] is consistent with the theory of complex adaptive systems. Complex adaptive systems are made up of heterogeneous interacting elements and it is the relationships between these interacting elements which lead to potentially unexpected outcomes [37]. Public health interventions tend to operate within complex adaptive systems [38].

Social Norms Theory proposes that behaviour is influenced by perceptions of behavioural norms [39]. This may lead to the new behaviour becoming the social norm, thus changing the behaviour at the population level. Alternatively, social norms may make it harder for a new behaviour to diffuse and become the norm. It is therefore important to consider these interactions, rather than focusing solely upon the individual, to make more reasonable predictions.

Social Identity Theory proposes that each individual self-categorises based on their perceived membership of social groups (e.g., researcher, mother, runner) [40]. The extent to which they feel they share characteristics with other members will, in part, determine the influence of the group on their behaviours. The social connection to others affects health and wellbeing through these influences on behaviour, as well as directly through being able to count on social contacts (e.g., close connections in a running club may affect maintenance, frequency and length of exercise). These social connections are largely ignored when determining health-related outcomes within health economic models, yet their impact on mortality has been shown to be greater than obesity, blood pressure and physical inactivity [41]. Social Identity Theory and the accompanying empirical evidence suggests that the focus on the individual to improve population health and wellbeing is insufficient [40]. In addition, the theory suggests that *maintenance* of healthy behaviour changes will be facilitated when an individual shifts from an identity for which the previous behavioural pattern was central to an alternative identity more supportive of the new behaviour (e.g., smoker to non-smoker) [42].

The life course approach recognises that over a lifetime, individuals accumulate health losses and benefits, and that at key forks in the life course, such as becoming pregnant or beginning work, these can be magnified and substantially affect future trajectories [43]. Thus, interventions may be more effective if they are targeted at specific stages of the life course. The life course approach recognises that by making changes to the environment and social norms, inequalities may be reduced, which may impact on life course trajectories, and this could benefit the whole population and future generations [44].

Ecological frameworks have been developed which draw upon multiple theories to combine both individual and environmental influences for different health-related behaviours [45]. These frameworks recognise the interactions of influences across levels; however, they do not generally include quantifiable constructs. In public health it has long been recognised that interventions work best if they are provided at multiple levels: the individual, the community and the population [46]. This has been best demonstrated for smoking policy which has been highly successful during the past five decades [45]; for example, stop smoking services provided to individuals, training programs for practitioners at the community level and a ban in public places at the population level [47]. It is important to consider the interaction between individual level factors and changes in the broader social and environmental context. The structure of the system is a key driver of its behaviour [37, 48]. We therefore need to be able to assess the potential impacts of changing the environment in addition to being able to assess individual-level interventions. Health economic modelling can provide a method for comparing and combining these interventions within one framework to explore their impacts, if the broader contexts that influence behaviour are incorporated within the models.

### Summary of key influences on behaviour

**Fig. 2:**
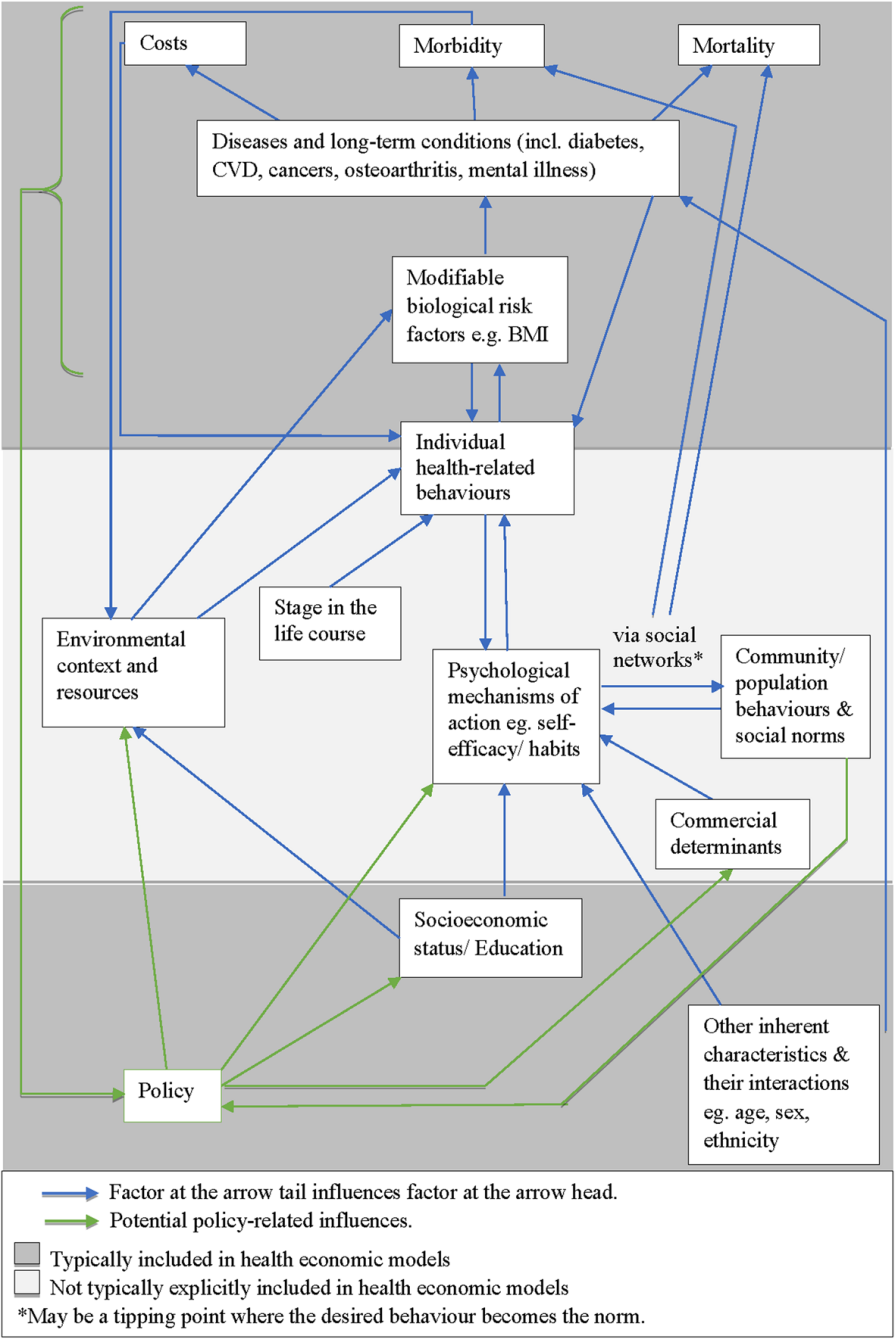
Influences on health-related behaviours and outcomes

Figure 2 summarises key influences on health-related behaviours and health economic outcomes as discussed in the above sections. In Fig. 2, those elements within the dark grey shading are typically included within health economic models of public health interventions, whilst those within the light grey shading are not [49–53]. Individual health-related behaviours span both the light and dark grey shading because they are *sometimes* explicitly included. There are copious amounts of information about the influences on behaviour from psychology, sociology and behavioural economics; however, understanding what evidence, theories and methods are useful (or not) for health economic modelling is not feasible within an applied research project, particularly if it is undertaken within the timeframes of a decision-making process.

### Aim of the paper

The primary aim of this paper is to draw on psychology, sociology, behavioural economics, complexity science and health economics to propose a practical toolbox of methods for incorporating the influences on behaviour into health economic models. For each method, we provide a description, an expert consensus on the circumstances when they could be used, how the method can bring behavioural influences into the model, the minimum resource requirements and key references. This will provide a useful resource for modellers to identify and incorporate appropriate methods and behavioural theory within health economic models of public health interventions, to better inform resource allocation decisions.

However, there are weaknesses in current behavioural theories, methods and the data available to inform such models (see Background). Thus, we also aim to set out an agenda for further research for both health economic modellers and behavioural scientists. Since this work is multidisciplinary, a glossary is provided in Supplementary Material (“Glossary”), to clarify technical terminology. This paper is accompanied by a website which provides a more interactive version of the toolbox [54].

## Methods

A consensus approach between experts from multiple disciplines was used to progress current methods. First, our multidisciplinary core team (HS, MK, NG, FS, RCP) undertook a literature review to identify and review all existing methods to incorporate health-related behaviour within simulation models across disciplines [55]. Based upon this, we developed a draft methods toolbox for incorporating behavioural theory within health economic models. We used an existing health economic model of diabetes prevention [8] initially to test and develop the toolbox conceptually. We used an agent-based model which investigated how behavioural theory can be used to predict population-level alcohol consumption patterns [56] to consider what would be necessary to develop such a simulation into a health economic model. Both are described in Supplementary material (“The School for Public Health Research (SPHR) diabetes prevention model” and “The agent-based model of alcohol consumption” respectively). We used these models because they were developed by some of the co-authors, so key elements of the model development process were known.

The core group was augmented by a wider group that covered a broader range of multidisciplinary expertise. We held a one-day in-person workshop with all 20 co-authors in February 2023 to develop the toolbox further. The multidisciplinary experts, based in the United Kingdom and Germany, were chosen according to their input into behavioural/ social science training courses, authoring key texts in their field or identification from the core multidisciplinary group as being a leader in their field. The group includes health economic modellers, health psychologists, behavioural and public health researchers, sociologists, behavioural economists, complexity scientists and agent-based modellers, a member of the patient and public involvement group, and policy makers. We circulated an early draft of this paper before the workshop. Within the workshop, we discussed how behavioural/ social science can inform and be informed by health economic models, the general structure of the paper, the potential barriers to the methods being used, and an agenda for future research. We used multidisciplinary breakout groups to discuss topics in more depth, followed by sharing key points and whole group discussions. Each breakout group had a designated note taker and a worksheet for all other authors to contribute notes. We narratively shared the outcomes of the workshop with the whole group and updated the toolbox accordingly. We subsequently collaboratively and iteratively developed improved versions of the toolbox, until qualitative agreement was reached between all authors.

We used the Behaviour Change Wheel [27] (described in the Background) to set out the requirements of interventions to encourage modellers to use behavioural theory within health economic models (see Supplementary Material, “A COM-B analysis of the use of a toolbox by modellers to incorporate the influences of behaviour into health economic models”). The content of this paper was designed to meet some of these requirements, there is an accompanying website to make the toolbox more accessible [54], and the remainder of these requirements are described within the agenda for further research (see Discussion). The ‘Results’ section of the paper sets out the toolbox, which is made up of a decision framework for choosing which methods might be appropriate (Table 1), a tabulated summary description of all the methods included in Table 1 (Table 2) and a full narrative description of each method and when to use (with each numbered heading describing a different method). The intention is that the user will employ the decision algorithm (Table 1) to make decisions about appropriate methods and then access the summary table (Table 2) and the narrative description to find out more about the appropriate methods as needed.

**Table 1:** Incorporating influences on Behaviour into Public Health Economic Modelling (PHEM-B) decision framework [v1.0]

				Record decision (and reason if applicable)
*Part 1) Always consider whether the following are feasible to understand the problem and for choosing and describing interventions:*				
Step A	Collaboration between health economic modellers and behavioural/ social scientists throughout intervention development and evaluation			
Step B	Reviewing the literature for the behavioural theories used to develop the intervention(s) to understand the problem in behavioural terms and identify the influences on behaviour			
Step C	Application of behaviour change intervention ontology			
Step D	Behavioural systems mapping			
*Part 2) Developing and justifying the model structure*				
*Characteristics of the behaviour(s) of interest:*		**Yes**	**No**	
Step E	(i) Are 2 or more types of behaviours likely to influence each other so that they will affect outcomes of interest more than the simple addition of outcomes associated with each behaviour (e.g., smoking & alcohol consumption)?	Move to (Eii)	Include the behaviour(s) of interest within the model. Move to (Fi)	
	(ii) Is there an individual level data set (preferably longitudinal and spatial) with the relevant behavioural outcomes reported or could these data be collected?	Consider econometric analyses. Move to (Fi)	Acknowledge the relationship & include the behaviour(s) of interest within the model. Move to (Fi)	
Step F	(i) Has the behaviour substantially changed in incidence or prevalence in the population over time?	Move to (Fii)	Move to (Gi)	
	(ii) Is there an individual level data set with the relevant behaviours and external factors reported over multiple time points or could this data be collected?	Consider statistical analyses. Move to (Gi)	Acknowledge these potential influences. Move to (Gi)	
Step G	(i) Based on steps (A) – (D), is the behaviour not substantially impacted by the behaviour of others (public or other types of stakeholders) or by the wider context OR is it clear that the interventions are likely to be cost-effective without accounting for these impacts?	Typical health economic model which does not incorporate interactions e.g., microsimulation. Move to (Hi)	Move to (Gii)	
	(ii) Are constraints around staff or other resources within the system important in affecting behaviours and outcomes of the people within the system?	Consider Discrete Event Simulation (DES). Move to (Hi)	Move to (Giii)	
	(iii) Is it likely that modelling the interactions between individuals and/or their access to places explicitly will be important in terms of population-level behaviour?	Consider Agent-Based Model (ABM). Move to (Hi)	Consider differential equation model. Move to (Hi)	
Step H	(i) Is an ABM being developed because the project team thinks that social network influences impact behaviours/ outcomes substantially?	Move to (Hii)	Move to (Ii)	
	(ii) Has relevant social network data collection and/or analysis been undertaken or is primary network data collection on the social network feasible?	Consider social network analysis. Move to (Ii)	Consider ABM incorporating abstract network e.g. scale free. Move to (Ii)	
*Type of question being assessed within the model:*				
Step I	(i) Do decision makers want to assess the impacts of increasing access to place (the built or natural environment)?	Move to (Iii)	Move to (Ji)	
	(ii) Is there data showing an interaction between access to place and behaviour or could this data be collected?	Consider ABM with spatial analysis. Move to (Ji)	Inform decision makers there is insufficient data to inform this intervention targeting. Move to (Ji)	
Step J	(i) Do decision makers want to assess the impact of interventions affecting behavioural mechanisms? e.g., level of motivation of the modelled individuals	Move to (Jii)	Move to (Ki)	
	(ii) Are relevant individual variables (e.g., a measure of motivation or capability) reported by the intervention study at multiple time points or could this data be collected?	Consider theory-informed statistical analyses. Move to (Ki)	Inform decision makers there is insufficient data to quantify behavioural theory. Move to (Ki)	
Step K	(i) Do decision makers want to assess an intervention which alters supply or demand of a good or service?	Move to (Kii)	Move to (Li)	
	(ii) Is there or could there be data on price elasticities?	Consider econometric analyses. Move to (Li)	Inform decision makers of the lack of data and consider undertaking exploratory analyses. Move to (Li)	
Step L	(i) Is individual level data available/ possible to collect on the behaviours and their influences for the intervention(s) and comparator over multiple time points?	Consider theory-informed statistical analyses	Review behaviour maintenance studies. Consider expert elicitation or qualitative research/ process tracing

**Table 2:** The 12 methods suggested within the PHEM-B toolbox v1.0 for modelling behaviour within health economic models

Method/ approach	What is it?	When and why use it for incorporating the influences on behaviour into the model?	How can the method/ approach bring behavioural influences into the model?	Which minimum data/ input are required?	Key references
1.Collaboration between health economic modellers and behavioural/social scientists throughout intervention development, delivery and evaluation	Early health economic modelling can inform when it is worthwhile to undertake a study of intervention effectiveness and relevant data collection. Behavioural/ social scientists can facilitate the collection and understanding of useful evidence and inform model assumptions, including beyond study follow up	Always consider collaboration throughout intervention development, delivery and evaluation to help inform policy efficiently	Behavioural/ social scientists can bring their expertise of theoretical assumptions, behavioural mechanisms and how they expect interventions to work over the long term. Health economic modellers helping to inform data collection will increase the feasibility of incorporating behavioural influences into models	An interdisciplinary network	Skivington et al. [1] Meier et al. [57] Bates et al. [58] West et al. [59]
2.Reviewing the literature for behavioural theories used to develop interventions and behaviour change/ maintenance studies	Reviewing the literature to understand the mechanisms of action of the interventions, including identifying existing theory of change models and behaviour change/ maintenance studies	Consider this to help understand the problem, including relevant theory and the longer term impacts, and to help choose interventions	Existing theories of the intervention mechanisms can be incorporated into the behavioural systems map which can then be used to inform model assumptions. If sufficient data on behavioural change and maintenance is identified, it could be used directly, or to inform elicitation, or as a calibration target	Information specialist. Discussions with policy makers/ stakeholders, and behavioural scientists	Skivington et al. [1] Michie et al. [27] Kwasnicka et al. [60] Madigan et al. [61]
3.Application of behaviour change intervention ontology	An ontology to provide consistent language within which to describe interventions (e.g., mode of delivery, setting, behaviour change techniques, mechanisms of action)	Consider this to describe interventions to model. Help with combining interventions appropriately within meta-analyses. Can be used to understand potential interaction effects	Describes what the interventions are made up of using consistent language, which allows appropriate delivery, evaluation, replication and assessment of fidelity	Access to the ontology, which is all open source	Michie et al. [62] [63]
4.Behavioural systems mapping	Diagrammatically representing relationships between actors, influences on behaviour and behaviour. Can be developed within a participatory process. Whilst systems mapping is well established, incorporating behavioural influences into these maps is novel	Consider this to help understand the problem, including relevant theory, choose interventions, inform statistical analyses and assumptions beyond study follow up	Macro and micro behavioural influences can be explicitly mapped out within a shared process across all stakeholders so that these can be discussed. This map can then be used to inform which behavioural influences are included within the health economic model and how they are included	Expert input and secondary literature	Hale et al. [64] Craven [65] Allender et al. [66]
5.Agent based modelling (ABM)	Individual-level modelling approach that allows for interaction between individuals AND with their environment	If there is evidence that social networks or environmental factors are important contributors of behaviour adoption or maintenance AND several interventions are being compared across multiple levels OR intervention targeting is an option	Agents within the model can be given rules about their behaviour dependent upon their characteristics, their past behaviours, psychological variables (e.g., motivation, capability) and/ or their interactions with others and the environment	Individual level data of the population with variables of interest; evidence to inform the decision rules of the agents	Gilbert [67] Giabbanelli, Crutzen [68] Vu [69]
6.Differential equation modelling	Differential equations to capture rates of change between variables	For describing the dynamics within a population across a complex system when interactions between individuals do not need to explicitly be captured. Can also be used alongside an individual level model to represent macro level (behavioural) changes if they are not explained by the included micro level variables	Differential equations can capture the relationship between the influences on behaviour and the behaviour of a cohort	Evidence about the relationships within the complex system (which may be qualitative & quantitative)	Occhipinti et al. [70] Breeze et al. [71]
7.Social network analysis	The specification of a social or information network using lines (relationships) and nodes (individuals/ organisations) to assess the impact of these networks on outcomes	If an ABM is being developed due to social influences being important and when resources allow	Incorporating social network analysis within an ABM enables individuals to influence others’ behaviour(s)	Identification of existing analyses OR data containing relationships between individuals and the outcomes of interest OR primary research	Knoke, Yang [72] Badham et al. [73] Smith, Burow [74] Snijders [75]
8.Geographical Information System (GIS) analysis to incorporate into an ABM	The specification of spatial elements in a population, e.g., green spaces or food outlets	An intervention being assessed is about access to certain places, there is substantial interaction between an element of the environment and behaviour upon which there is some evidence	Incorporating spatial data into an ABM allows the physical environment to influence individuals’ behaviour(s)	GIS (spatial) data	Crooks et al. [76] Crols, Malleson [77]
9.Discrete event simulation	An individual level simulation approach where individuals interact with the system through a series of events, which may be resource constrained	When behaviour and outcomes are influenced by physical resource constraints	Behavioural theory could be incorporated within a DES to model the staff which constrain the system or the individuals who use the system	Timing of key events in the system, quantity of constrained resources, & ideally individual level data	Robinson [78] Karnon et al. [79] Brailsford et al. [80]
10.Theory-informed statistical and econometric analyses	Statistical techniques to assess relationships between variables based on relevant theory	To model the influence of behaviours upon each other (e.g., smoking and alcohol consumption) if the behaviours impact the same outcomes of interest OR when population behaviour has been shown to change over time OR to inform assumptions beyond study follow up when individual level data is available at multiple time points	Either (a) the influence of behaviours upon each other can be incorporated, or (b) external factors that may influence the behaviours of different groups of the population can be incorporated appropriately; or (c) uses behavioural or economic theory to make predictions about behaviours over time which can be incorporated within the health economic model	Individual level data set with relevant variables	Sullivan [81] Bianconcini, Bollen [82] Bates [83] Bell [84]
11.Expert elicitation	Quantifying subjective and implicit expert knowledge	To inform the long-term model parameters when there is a lack of quantitative data by utilising expertise of behavioural scientists	By quantifying expert knowledge of the influences on the relevant behaviour(s) over time	Ideally, input from at least five relevant experts	O'Hagan [85] Bojke et al. [86]
12.Qualitative research/ process tracing	Collection and analysis of data from observational studies, interviews or focus groups. Process tracing involves theorising why an intervention works and then collecting data to test and amend the theory within an iterative process using a set of individual cases	To provide a rich understanding of how and why individuals behave in the way they do, to understand what to measure, to inform model assumptions about behaviours, including informing heuristics that individuals follow. Process tracing can be used to describe the casual mechanisms between interventions and the behaviour of interest when it is not well understood	Provides a richer understanding of the influences on behaviour, can inform data collection, and can improve theory associated with the causal mechanisms of interventions which can inform the model assumptions	Review existing studies or primary data collection and analysis	Tubaro, Casilli [87] Beach, Pedersen [88] Flemming et al. [89]

## Results

A decision framework to help modellers incorporate the influences on behaviour within their health economic models of public health interventions is shown in Table 1 below. This is divided into 2 main sections: Part 1 includes approaches that should always be considered if feasible for understanding the problem and for choosing and describing interventions. Part 2 includes approaches for developing the model structure, dependent on the characteristics of the behaviour(s) of interest and the type of question being assessed within the model. The user should consider each question in Part 2 in turn, and methods are not mutually exclusive, so for example it may be appropriate to use a combination of agent-based modelling (ABM) and econometric analyses. Table 2 shows summary information about each of the methods included within Table 1, including what the method is, when and why it could be used, how it brings behavioural influences into the model, minimum data or input required, and key references about the method. The use of each method will be dependent upon practical considerations, in particular the time available within the decision-making process. However, since this would be a factor for all the methods outlined, it has not been explicitly stated for each one. The methods are described in detail within the remainder of the paper. Many of the methods are highly resource intensive, and some approaches for reducing this, where possible, are outlined.

The combination of the decision framework and the description of the methods provides a toolbox for modellers to incorporate the Influences on Behaviour into Public Health Economic Modelling (‘the PHEM-B toolbox v1.0’). It is worth noting that the aim is not to identify all methods for public health economic modelling, but those which would allow the influences on behaviour to be incorporated to better predict the impacts of public health interventions.

### Method 1: Collaboration between health economic modellers and behavioural/ social scientists throughout

Collaboration is about working together towards a shared goal to produce better outcomes than could be achieved individually. There would be a benefit to greater collaboration between behavioural and social scientists and health economic modellers in a cyclical process of intervention development and evaluation [57, 58]. This would align with the latest guidance for developing and evaluating complex interventions [1]. Collaboration could enable better understanding by health economic modellers and behavioural scientists about which elements of their research are important for achieving shared goals and how they can inform each other. Early health economic model development/ input may lead to the conclusion that it is not worthwhile to study the effectiveness of a public health intervention, or it could inform useful data collection for evaluation. Psychologists, sociologists and/ or behavioural economists could help modellers to understand the evidence, the behavioural theory used to develop interventions, and to help inform assumptions beyond study follow up where no or limited data exist.

The APEASE criteria has been developed for assessing interventions at any stage of development or evaluation [59, 90, 91]. This comprises Acceptability, Practicability, Effectiveness (and cost-effectiveness), Affordability, Spill-over effects, and Equity, and if any of these are not deemed by stakeholders to be met then the intervention should not be considered. Throughout this paper, stakeholders include members of the public affected by the interventions, people delivering the interventions and commissioners of the interventions. The APEASE criteria could be used to develop shared goals for collaboration. Within a health economic model, Effectiveness, Affordability, Side-effects and Equity can be explored, whilst it would not be worthwhile assessing interventions within a health economic model if they were not considered to be acceptable or practical by stakeholders.

### Method 2: Reviewing the literature for (a) theories used to develop the interventions and (b) behaviour maintenance studies

Teams evaluating the effectiveness and cost-effectiveness of a set of interventions could identify which theories were applied (if any) when interventions were developed, if these are reported. The interventions and their influences could then be added to a behavioural systems map (see Method 4). This information can be presented to and discussed with policy makers and other relevant stakeholders to help select interventions for evaluation. Behavioural theory could be qualitatively utilised to inform long term assumptions within both cohort and individual level models. Where intervention studies present theory of change diagrams (identifying an intervention’s impact, outcomes, outputs, activities, and inputs and describing why interventions will create which outcomes) these could be used to inform long term assumptions.

Most existing theories and intervention studies focus upon behaviour change rather than behaviour maintenance (i.e. sustained behaviour over more than 6 months) [60]. The influences on behaviour change should not be assumed to be the same as those affecting behaviour maintenance. For the prevention of many non-communicable diseases, the goal is to *maintain* healthier behaviour over the longer term, to impact long term health outcomes. Different interventions are likely to work in different ways, and in different populations, and hence some interventions may be more likely to assist behavioural maintenance rather than others. Most studies report only the effectiveness of the interventions over 6 or 12 months. There are a small number of intervention studies which are designed to capture behaviour maintenance outcomes, as well as some meta-analyses of these studies, mainly for physical activity maintenance [16, 61, 92–95]. A literature search for relevant behaviour maintenance studies could be undertaken. It may be possible to apply these studies to inform model predictions; however, outcomes are still relatively short term (generally, a maximum of three years of data), and only average effects are reported. These data could be used directly, or to inform elicitation, or as a calibration target. Information specialist input would be required to design these literature searches.

### Method 3: Application of the behaviour change intervention ontology to describe interventions to model

The behaviour change intervention ontology (BCIO) [63] has been developed to provide consistent language within which to describe interventions, including the mode of delivery, setting, source, schedule and dose, population, method of engagement, style of delivery, behaviour change techniques (the content of the interventions), mechanisms of action (how the interventions work), human behaviour and intervention fidelity [62]. The TIDieR Checklist has previously been developed to improve reporting of interventions [96], which includes many of these elements and may be less resource- intensive. However, the benefit of the BCIO is that for each label in the ontology there is a unique ID number. This can facilitate consistency between intervention development and evaluation, and across models and theories. It can also enable the synthesis of similar interventions within meta-analysis which can be computer automated, which can be used within the health economic modelling. Specifying the detail of the interventions in this way could also help in informing the behavioural systems map and understanding the potential long-term impacts of the interventions. It is yet to be determined whether the BCIO fully captures all elements of interventions, for example behavioural economic interventions; however, the intention is that the ontology will be updated accordingly within future versions when elements are found to be missing.

Where a set of interventions are being assessed in combination in the model, but effectiveness evidence is only available for the individual interventions, the content of the interventions and behavioural theory could be used to understand the mechanisms of action. If they operate through completely different mechanisms because their content is different, then assuming an additive effect may be appropriate; and if some overlap, then a multiplicative effect. In addition, based upon the themes identified for behaviour maintenance [60], maintenance motivation, resources, environmental support and self-efficacy are all required for behaviour maintenance. Thus, interventions which impact all of these, when combined, may substantially increase the probability of behaviour maintenance compared with any of the interventions alone. Since within a health economic model it is necessary to predict the impact of the interventions over the long term, this could be used to help choose plausible long-term assumptions of intervention combinations.

### Method 4: Behavioural system mapping

Behavioural systems mapping is a developing method which uses systems thinking to diagrammatically represent relationships between the behaviour(s) of interest, interventions, actors, and the influences on behaviour, for the purpose of helping decision-makers or other stakeholders to understand systemic influences on behaviour(s) of interest [64] and the implications for interventions. Squires et al. [38] proposed the use of systems mapping to understand the problem needing to be modelled prior to developing the health economic model to understand relationships between factors and to help decide what to include and exclude from the model, but this framework did not consider influences on behaviour explicitly. To be able to model what may happen over time, modellers need to gain an understanding of the influences on the behaviour(s) and the impact of the intervention(s) upon the behaviour(s). Figure 2 within this paper and formal behavioural theories, alongside input from behavioural scientists, could be used to inform the development of a behavioural systems map. These could include both micro variables which describe individual attributes (e.g., motivation) and macro elements which describe aggregate level phenomenon (e.g., social network structures), and the potentially causal relationships between them. If the policy makers are aiming to reduce inequalities and/or consider intersectionality, then it will be important to consider the different influences impacting upon relevant groups of interest.

Describing these influences within a systems map will provide a tool for communication between behavioural and social scientists and health economic modellers and other stakeholders, increasing the understanding of the problem and facilitating the development of a useful model (see Supplementary material, “The School for Public Health Research (SPHR) diabetes prevention model”, for an example). Behavioural systems mapping can provide a transparent process through which to make decisions about what is included (or not) within the health economic model. It can also facilitate discussion about the social value judgements which may be made. The outcomes reported by intervention studies may limit what quantitative analyses can be done within the model, for example if only body mass index is reported rather than physical activity or dietary outcomes or the mechanisms of action associated with these. In addition, studies often do not report outcomes according to attributes associated with inequality (for example, by ethnic minority or gender) or intersectionality (for example, by Asian women). In these cases, behavioural systems mapping may provide a qualitative understanding of the micro and macro level influences, which can help to inform what to include and exclude from the model, assumptions beyond study follow up, and what further data collection may be useful.

Behavioural systems maps can be developed from participatory stakeholder input [66], and/or other sources including qualitative research such as interviews or quantitative analysis based on surveys [65]. These could either be developed at an early stage and could help to inform the choice of interventions to assess, or if the interventions have already been chosen, the theory used to develop the interventions (where available) could be incorporated.

### Method 5: Agent based modelling

Agent-based modelling (ABM) is an individual-level simulation approach where the agents are given rules about their interactions with each other and their environment, which may depend on their individual characteristics [67]. It differs from typical microsimulation modelling because it is possible to incorporate interactions and feedback by explicitly modelling social networks and/ or the physical (built and natural) environment [71]. These interactions enable the analysis of emergent properties and tipping points where a new behaviour becomes the social norm over time. Whilst it is possible to model proximity to place within typical microsimulation models, more complex rules about the influence of social networks and the physical environment upon behaviour can be incorporated, as well as the possibility of changes to the environment as the behaviour of individuals changes. Individuals can be given different rules according to their psychological attributes or individual characteristics, which enables the impact of influences upon people’s behaviour to vary. In turn, this allows for more nuanced understanding of the equity impact of different interventions, including potential for disaggregation of results by intersectional subgroups. It is also possible to model multiple types of agents, for example, consumers and tobacco companies. There may be interactions between the behaviours of these different types of actors which lead to unexpected outcomes. It may be feasible to validate emergent population level impacts of the interventions with data.

If feasible given the resources available, ABMs are preferable over other modelling approaches when one or more of the following holds: (i) It would be useful to explore the impact of interactions between the behaviours of different stakeholders, such as the public and the tobacco industry; (ii) The model aims to assess the cost-effectiveness of interventions about access to places affecting public health, such as green spaces or food outlets; AND there is evidence of substantial interaction between the environment and behaviour; or (iii) There is evidence that social networks will substantially affect relevant outcomes (beyond what was reported in the intervention studies), which may include impacts on health inequalities AND it is unclear whether the interventions would be cost-effective without accounting for these additional impacts.

Models should be as simple as possible to capture the key drivers of the outcomes, and hence it is important to weigh up the benefits of developing an ABM and including the influences on behaviour within the model versus the time and resources required to build such a model. In some cases, there is very little evidence about the costs and/or effects of public health interventions in changing short term outcomes even at an aggregate level [3, 97], hence more evidence needs to be collected before more complex models could be usefully parameterised for prediction. In addition, models are only useful if they are credible to stakeholders and policy makers. It may be that increased complexity may decrease credibility, and hence it will be beneficial to co-design models with stakeholders, discussing alternative modelling options, as well as reporting modelling methods and assumptions transparently [98].

ABMs require individual-level data about the population containing the key variables of interest and some evidence to inform the rules of the agents. One of the major advantages of ABM is its flexibility; any of the influences on behaviour outlined within Fig. 2 could be incorporated in an ABM if required, using a range of data, from qualitative to quantitative. Rules for the agents could be informed by (a combination of) behavioural systems mapping, qualitative research, heuristics, behavioural theory, statistical or econometric analyses, or secondary literature.

A few attempts have been made to incorporate psychological theory within ABMs of public health interventions [55]. These studies demonstrate the potential, but also the substantial time, skill and data requirements for such evaluations. Little justification is usually provided for the theory chosen, though some studies have undertaken systematic reviews [99] and conceptual modelling with expert input to identify the most appropriate theorical basis [100]. The only behavioural theory which has been quantified within a simulation model to date that considers behaviour maintenance explicitly is the Transtheoretical Model of Change [100, 101]. This involves the stages: Precontemplation; Contemplation; Preparation; Action; and Maintenance, and it acknowledges the possibility of relapse. It has, however, been heavily criticised in the health psychology literature because it ignores habits and situational determinants of behaviour [26]. Buckley et al. [102] used dual process theory within a simulation model of alcohol consumption, with a habitual pathway and an intentional pathway, and a recognition that new habits in terms of alcohol consumption could be formed—a key determinant of behaviour maintenance [60]. Other simulation models that have quantified behavioural theory have updated the parameters at each time step but assumed the same mechanisms of behaviour maintenance as behaviour change [55]. The developers of the COM-B model have considered sustained behaviour change and suggest that changes to capability, opportunity and motivation must be mutually reinforcing for behaviour to be maintained [103].

It may be possible to utilise (or adapt) existing agent-based models of public health behaviours which use empirical data, and then incorporate the costs and effects of the interventions being assessed; however, many ABMs are developed to explore a population-level phenomenon over a short timeframe, so may not be easily modifiable for the purposes of health economic modelling (see the alcohol consumption example in the Supplementary Material, “The agent-based model of alcohol consumption”). Models built in modular form can be combined and reused, particularly if shared using open-source software. This would make it more feasible to build ABMs within the constraints of a policy making process. It would be possible to modify an existing individual level health economic model to incorporate the effects of social networks or the environment (see the diabetes prevention example in Supplementary material, “The School for Public Health Research (SPHR) diabetes prevention model”). A software architecture has recently been developed for mechanism-based social system modelling to incorporate behavioural and social theories within ABM [69]. Changes in macro level behaviours are generally determined by the interactions between micro level behaviours and macro level behaviours; however, it is also possible to use differential equations to represent macro level (behavioural) changes if they are not explained by the included micro level variables.

### Method 6: Differential equation modelling

Differential equation models, including system dynamics models, use differential equations to capture the rates of change of a set of variables and the relationships between them [37]. These models are useful for describing the dynamics within the population, which may include how behaviour is influenced within a complex system [70, 71]. However, it becomes more challenging to utilise this modelling method alone when decision makers would like to consider outcomes for many sociodemographic indicators, or when proximity to place is important in terms of behaviours. Differential equation models alone also cannot capture interactions between individuals in a way that would likely be useful for understanding the effect of interventions as they cannot explicitly capture different social network structures.

### Method 7: Social network analysis

Social network analysis involves the collection and analysis of survey data about with whom individuals interact and their relationships, so that social or information networks can be specified using lines (relationships) and nodes (individuals/ organisations) [72]. At the same time behavioural outcomes of interest can be collected, such as alcohol consumption. The impact of the social networks upon behaviours can then be assessed.

It may be that intervention studies are undertaken within a population or community where the impacts of social networks are already (implicitly) captured, for example studies assessing the smoking ban in public places. Assuming additional impacts of the interventions due to social networks in this situation could lead to double counting if study follow up is sufficient. It is therefore important to understand the sample included within the intervention studies of interest; if it reflects the population and follow up is substantial then it may not be appropriate or worthwhile incorporating social networks explicitly. However, if social influences on behaviour would not have been captured by the intervention studies, then social network analysis could be considered. Undertaking social network analysis has the advantage that the relationship between the social network and the behaviour(s) of interest can be informed by the data collected.

Collecting data from the full network is ideal; however, practically this may not be possible. Egocentric network analysis, which can be done by collecting relationship data from individuals (or ‘egos’) from a sample of the population who may or may not be connected, may be more feasible and has been shown to provide reasonable results compared with a full network [74]. Most social network analysis assumes that social relationships are constant. Stochastic actor-based models for network dynamics allow social relationships to evolve over time as they would in practice [75]. This approach could be considered when an intervention might change social relationships, for example, if university students are given interventions to reduce binge drinking. It requires social network data for at least two time points, although preferably more.

A literature search for social network analysis associated with the behaviours of interest is recommended to first assess the benefits of including social network analysis and second for model parameterisation. Studies exist for some behaviours and outcomes which could be utilised [104, 105] if the population is relevant. If there are no existing analyses and there are insufficient resources to undertake social network analysis, random networks, small world networks (all individuals linked by a small number of nodes) or scale free network (most people have a small number of connections, whilst a few have a large number) could be used within an ABM, and there are existing software packages to do this. However, these networks make ties at random or conditional on some characteristics and might not reflect the real social network, and assumptions are required about how individuals influence each other’s behaviours.

### Method 8: Geographical Information System (GIS)

A GIS is essentially a spatial database that holds specifications of the spatial elements of a population (who lives where), or features of the physical landscape, such as roads or green spaces, or the built environment, such as food outlets [76]. Within ABMs, GIS information is often represented using one of two approaches; (i) raster data (that is, a large number of square cells, and then attributes are assigned to them; typically applied to environmental applications) or (ii) vector data (points, lines and polygons). The latter is the more ‘popular’ choice as this format (commonly termed shapefiles) allows the physical environment of roads, buildings and other urban features to be readily represented in fine granularity. With recent developments in popular platforms, such as Netlogo or GeoMason, it is possible to import shapefile layers directly into the platform and ABM simulations run directly within them [77].

Integrating GIS within ABMs may be useful for health economic modelling if an intervention being assessed is about access to certain places and there is substantial interaction between an element of the physical environment and behaviour, upon which there is some evidence. It could help to explore where places should be located to maximise cost-effectiveness and/ or reduce inequalities. Note, it is possible for ABMs to incorporate more abstract geographical elements in the absence of GIS to inform such policy decisions [106].

Undertaking GIS analysis requires geo-referenced data, many of which are online and open-source. These include OpenStreetMap (contains map data including roads, trails, cafes and railway stations) and Natural Earth Data (contains counties and points of interest) (see Crooks et al. [76] for a discussion of the different formats). There is also open-source software allowing individuals to create, edit, analyse and visualise the geographical data, which includes Quantum GIS and GRASS software. In addition, many of the platforms available for ABMs have the capability to process GIS data. For example, within NetLogo there is a GIS extension, and it is possible to import both raster and vector data [76].

### Method 9: Discrete Event Simulation (DES)

DES is an individual level simulation approach where individuals interact with the system through a series of events, which may be resource constrained [78]. Within resource-constrained DES, queues can build up due to insufficient resources within the system, which can lead to long wait times. For health economic modelling, it is generally assumed that it would be feasible to implement interventions within the current system, with no additional physical resource requirements [79]. However, this may not always be the case, and limited resources within a system could affect individual behaviour and outcomes. For example, patients may decide not to utilise stop smoking services because the wait time is too long, particularly if they are less motivated to quit. Decision makers may want to evaluate the impacts of changing the physical resources, for example greater access to stop smoking services. Human behavioural theory could be used within a DES to model the staff that constrain the system (for example, staff may have long periods of sickness absence due to overwork which could lead to longer waiting lists and more overwork), or the individuals who use the system (for example, patients’ previous screening attendance may be a good predictor of future screening attendance). Thus, DES has advantages over other approaches for incorporating the influences on behaviour when behaviour and outcomes are influenced by physical resource constraints.

Currently, very few health economic DES models have been developed which incorporate behavioural theory [80]. There are lots of software options for DES, most of which provide a visual interface (e.g. Simul8 and Arena), which are helpful for sharing with stakeholders. DES requires information about timing of key events in the system, including arrival times, and quantity of constrained resources. Personal characteristics and psychological variables which would affect behaviour and outcomes of the people within the system would also ideally be incorporated.

### Method 10: Theory-informed statistical and econometric analyses

Statistical analyses describe relationships between a set of variables, with econometric analyses involving the application of economic theory to formulate those relationships. There is a plethora of statistical and econometric methods available and so we do not attempt to cover them all here. Instead, the circumstances when these methods might be useful for incorporating the influences on behaviour into health economic models are described and references to papers discussing the key methods are provided. For any statistical approach chosen, these should be informed by available theory to have more confidence in results and avoid overfitting to the data.

Statistical methods could be used for incorporating the influences on behaviour within health economic models for the following (non-mutually exclusive) reasons:

1. To model the relationships between behaviours which influence each other;
2. To model the long-term impact of interventions upon behaviours;
3. To model population-level behaviours over time.

Econometric methods for modelling the relationships between behaviours could be considered when behaviours are highly likely to influence each other and the behaviours affect the same outcomes of interest to decision makers, for example, smoking and alcohol consumption [81]. Behaviours may be complements (decreasing one will decrease the other), substitutes (decreasing one will increase the other) or have no influence on each other. A longitudinal or repeated cross-sectional individual level data set with relevant variables and expertise in econometric analyses would be required to infer causal relationships between behaviours. The econometric analysis should highlight and discuss any necessary assumptions, especially those that cannot be tested.

Statistical analyses could be used to estimate the trajectories of behaviour and the impact of interventions upon a behavioural outcome where it is challenging to directly measure the longer term effects. Bianconcini and Bollen [82] describe a set of methods which can each be considered special cases of the Latent Variable-Autoregressive Latent Trajectory Model for longitudinal data analysis. Quantification of behavioural theories would be useful within a health economic model if: (i) the intervention aimed to change at least one variable within the theory (e.g., self-efficacy or social influences); or (ii) policy makers would like to explore targeting interventions at individuals with certain levels of a variable within a theory (e.g., level of physical resources or intention to quit). It should be noted that there is little time lag between changes in the mechanisms of action and behaviour, meaning it is not possible to predict future behaviour from current mechanisms of action in the same way that potential future disease can be predicted from current risk factors. In addition, data for the variables (e.g. a measure of motivation) are often not measured or reported within intervention studies. Increasingly, interventions may involve individuals reporting regular psychological, behavioural, health and economic outcomes on mobile phone apps. The use of such devices can provide many data points from individuals receiving an intervention, and could cheaply provide maintenance phase data, which would allow the longer-term impacts of the interventions to be better understood. Economic demand theory can be applied for statistically modelling the relationship between price and consumption where an intervention changes either supply or demand (e.g., implementing a soft drink tax). However, uncertainties around taxation policy impacts and long-term prediction should be highlighted; for example, there may be differences in the size of price changes observed in the data and those used for taxation policy changes. It is also important to consider heterogeneity in these relationships where possible.

Population-level behaviours may change over time because of ageing or external factors that affect the whole population (e.g., economic crisis, shift in social norms). These may affect different age groups differently and there may be cohort effects where behaviour varies according to birth year. Age Period Cohort analysis aims to understand and disentangle these effects using statistical analyses [84]. This may be useful where behaviour has been shown to change over time and it is likely that a behaviour is affected by all three effects, for example smoking. The analysis requires a longitudinal or repeat cross-sectional individual level dataset, with the relevant behaviours, age and external factors reported.

### Method 11: Expert elicitation

Expert elicitation involves quantifying subjective and implicit expert knowledge and is useful when there is insufficient data available to quantify model parameters and their uncertainty [86]. Elicitation could be used to inform the parameters for the long-term model assumptions, particularly for the intervention effects, when there is a lack of quantitative data. Multiple experts should provide input [86], and these should include behavioural scientists. It is important to understand dependencies between elicited parameters (e.g., different points on a survival curve) so that the dependencies can be incorporated within the model explicitly [86].

In order to reduce bias, elicitation protocols should be followed [85]. Leading protocols include:

- the Delphi method, where individual judgements are made, a summary of all the individual judgements is shared, before one or more additional rounds of providing judgements, followed by group summaries, until these are mathematically aggregated;
- the Cooke protocol, where experts individually make judgements about uncertain quantities as well as quantities known to the researcher and then the uncertain judgements are weighted by their performance on the known quantities and mathematically aggregated; and
- the Sheffield Elicitation Framework (SHELF) protocol, where individual judgements are made and then these are discussed with the group, including the reasons for differing opinions, until a consensus judgement is made.

For all of these protocols, questions should be piloted to ensure they are valid, intuitive and clear [86]. There are existing software and tutorials available to facilitate elicitation [85].

### Method 12: Qualitative research/ process tracing

Qualitative research includes collecting and analysing data from observation, interviews or focus groups which can provide a richer understanding of how and why individuals behave in the way that they do in a set of hypothetical situations [87]. It can be used to inform model assumptions and may be able to provide more valid assumptions than those developed based on quantitative data alone. When making decisions about behaviours, people often use heuristics which are “strategies that ignore part of the information, with the goal of making decisions more quickly, frugally, and/or accurately than more complex methods” [29, 30]. For instance, within an ABM, a heuristic decision tree may be used, where alternative cues affecting a decision are taken sequentially in order of importance, termed the fast and frugal approach [76], and these could be informed by qualitative research.

Process tracing is another way of describing the mechanisms between a cause (e.g., the intervention) and an outcome (e.g., the behaviour of interest), by observing how the intervention works within a set of individual cases [88]. Theory about why there is a relationship between a cause and outcome is developed and empirical observational data is collected to test and amend the theory within an iterative process. This could then be incorporated within a health economic model. However, this is a resource intensive process, and the theory should only be generalised very cautiously beyond the population within which it is tested.

Existing qualitative studies may be reviewed [89] or primary qualitative data collection and analyses could be undertaken where feasible. Data could be collected from a diverse set of people from the target population, or qualitative research may be useful for filling gaps in knowledge about the influences on behaviour for an understudied high-risk subgroup.

## Discussion

### A new way of thinking

Health economic modelling should be founded in theory and use data to compare alternative interventions. Existing behavioural and social science is currently underutilised for making predictions within health economic models of public health interventions. A toolbox of methods for incorporating the influences on behaviour within health economic models has been developed and is made accessible via the accompanying website [54]. We anticipate that methods from the toolbox will be used in combination, and hybrid modelling approaches may be useful, including combining macro and micro level approaches. Within the behavioural and social sciences, theories and methods are constantly evolving, and it is anticipated that this toolbox will need to be updated as research within other disciplines develops.

There is much research being undertaken by behavioural scientists to develop public health interventions based upon behavioural theories and frameworks, but this is mostly not accessed by health economic modellers when evaluating interventions. In addition, health economic models are generally developed after an intervention has been tested and the data needed may not have been collected. There would be a benefit to greater collaboration between behavioural scientists and health economic modellers in a cyclical process of intervention development and evaluation, which could save costs and improve allocation of scarce resources.

Public health interventions operate within complex systems and as such it is essential to consider the importance of the interactions between individuals and with their context on predicting outcomes. Behavioural systems mapping can be used to understand these interactions and the potential relationships between interventions, psychological, social, biological and environmental mechanisms and behaviour, as well as for making transparent decisions about what to include and exclude from the health economic model and understanding the potential impacts of model simplifications [38]. Behavioural systems mapping could be useful for describing the different influences upon behaviour. It should be noted that complex models are not always necessary for modelling complex systems. For example, if an intervention is cost saving and has been shown to be effective, then it would not be necessary to develop such a model. In addition, there may not be sufficient time and/or data available. However, modelling complex systems can enable key influences on behaviour to be explored including the interactions between heterogenous individuals and their environment. When there is evidence of differential impacts according to relevant attributes, these models can be used to assess the impact of interventions on health inequalities. Whilst Big Data are becoming more accessible and may be useful to inform models, data alone cannot provide an understanding of all the relevant mechanisms and processes that are operating and should be accompanied by theory to make predictions.

It is important for policy makers to understand that it is typically not possible to predict, with any precision, long term outcomes within a complex system, and hence health economic models of such systems are unable to provide accurate cost-effectiveness estimates over a lifetime horizon for public health interventions. However, the process of model development and the model results can be informative in comparing and understanding different intervention options and facilitating decision making [107]. Health economic modellers should be clear in their reporting about the theories used, their assumptions, the limitations of the models and, the uncertainties within the model results. For ABMs and other individual-level simulations, there is a well-developed framework for model reporting [98].

Within health economic modelling, the main approach to quantifying uncertainty is probabilistic sensitivity analysis, where the uncertainty in model inputs is propagated through to model outputs [108]. It is recommended that structural uncertainties should be quantified within the probabilistic analysis where possible, and scenario analyses are run to explore alternative futures [109]. Within complex models, substantial uncertainties often exist within the model structures as well as the parameters, and it may not be feasible to quantify these. Machine learning has been used for exploring the impact of structural uncertainties, including comparing alternative behavioural theories [110]; however, this approach has not yet been used for health economic modelling. In addition, data may not exist for all variables of a behavioural theory. Calibration methods are likely to be required to inform unobserved parameters, within which uncertainty should be incorporated. Where possible, validation of each part of the model and the system level intermediate outcomes should be undertaken with different data from that used to build the model. Breeze et al. [71] provide a more detailed discussion of uncertainty analysis, validation and calibration within complex systems for economic evaluation; however further research is required in this area.

Developing modular models, where sections of code can be accessed as necessary, has the advantage of being able to be reused within other models, providing that code is shared within open-source software. This sharing between modellers can improve model building, verification, transparency and validation, as well as enabling faster model development. However, there are barriers to open code sharing which will need to be overcome [111, 112]. There are different types of open-source licensing that can stipulate certain conditions, for example limitations on commercial use. Code sharing alongside clear model reporting would increase the feasibility of using these more complex modelling methods within a resource-constrained decision-making process. It could also allow models of different behaviours to be combined so that the interactions between different behaviours can be incorporated where this is important.

We emphasise that this paper aims to understand the interventions being evaluated so that appropriate assumptions about the long-term impacts of the interventions can be made. Ideally, modellers would understand the types of intervention (access to place, price changes, targeting individuals with specific characteristics/ psychological variables, targeting ‘influencers’ within a social network) and the sorts of evidence about intervention effectiveness (outcomes being reported, before and after study, number of data points, length of follow up, individual level data) which could change decisions about the methods employed. If this is not possible, then the model will need to be flexible to these considerations.

### Agenda for further research

Currently, within most policy making arenas there is insufficient time and resources allocated to evaluating the cost-effectiveness of public health interventions, leading to very simple models of these complex systems being developed. Meanwhile, the studies of the interventions are very short term, not always clearly described, and with aggregate results presented. Therefore, there is a substantial further research agenda across fields to advance methods for incorporating the influences of behaviour into health economic models in order to better inform public health policy.

1. Develop collaborations between health economic modellers and behavioural/ social scientists to inform intervention development, to help understand at an earlier stage whether interventions are likely to be cost-effective and to ensure that useful outcomes for the health economic modelling are collected and reported. A process for working together effectively could be developed following the use of the toolbox within case studies. Health economic modellers could work with behavioural/ social scientists to understand how behaviour maintenance theories might best be utilised within health economic models, and what further research would be beneficial to improve long-term predictions of intervention effectiveness. The authors plan to set up a new network between modellers and behavioural scientists to encourage collaboration and to share resources.
2. Develop a consensus statement on the most appropriate behavioural theories for each health-related behaviour, ideally through collaboration between psychologists, sociologists and behavioural economists. Subsequently, develop and collect relevant standardised measures of behaviour and influences on behaviour, which use a consistent ontology. Collect longer term data where possible when evaluating the effectiveness of interventions. This could be done by using mobile phone apps or wearable sensors. Develop and test behaviour maintenance theories for different health-related behaviours.
3. Develop a suite of public health economic agent-based models which are built flexibly and reported open source, including coding, which would allow model reuse and adaptation. This would allow modellers who have limited resources and time within the decision-making process to build upon existing models. Standard social network structures and GIS data could be included which can be modified if both feasible and necessary. If these ABMs were built consistently across model behaviours, then they could link together if behaviours affect each other. Collaboration between health economic modellers and software engineers could improve model development efficiency and reuse. Exploiting recent advances in artificial intelligence may also facilitate this. Evaluate the benefits of the ABMs over standard health economic modelling approaches.
4. Develop methods for informing long term assumptions about intervention effectiveness. Test the appropriateness of developing expert panels and applying elicitation approaches to help inform structural assumptions and quantify parameters where there are no data. This could include lay people with relevant lived experience. Assess the feasibility of combining qualitative analysis with health economic modelling to inform behavioural assumptions. Explore the potential of utilising GP records (NHS digital) to assess the long-term effectiveness of interventions. Evaluate the benefits of these approaches.
5. Train modellers to utilise the new methods via short courses, webcasts, and workshops which would need to include an overview of the rationale and the methods, as well as demonstrating the use and outcomes with an example. Within the training, modellers could be given an opportunity to practice the methods using a simple example.

## Conclusions

Public health intervention studies often have short-term follow up and they operate within dynamically complex systems. To model beyond the study data, it is essential to understand the influences upon behaviour, including the social determinants of health and health-related behaviours. A toolbox of methods has been developed as a starting point to help modellers incorporate the influences on behaviour into health economic models of public health interventions. The toolbox sets out when and why each method would be appropriate, and the minimum resources required. It may not always be feasible or necessary to model the influences on behaviour explicitly, but it is essential to develop an understanding of the key influences. Collaboration is needed between health economic modellers and behavioural/ social scientists throughout the process of intervention development and evaluation to help inform policy efficiently, and to generate approaches for utilising behaviour maintenance theories within health economic models. Further research is needed to develop a suite of more robust health economic models of health-related behaviours, reported transparently, including open-source model code, which would allow model reuse and adaption.

## Supplementary Information

Supplementary Material 1.

## Data Availability

All data generated or analysed during this study are included in this published article [and its supplementary information files]. The PHEM-B toolbox is also available in an accompanying website [54].

## Abbreviations

ABM: Agent based model
BCIO: Behaviour Change Intervention Ontology
BMI: Body Mass Index
COM-B: Capability, Opportunity, Motivation-Behaviour
DES: Discrete Event Simulation
GIS: Geographical Information System
PHEM-B: Influences on Behaviour into Health Economic Models of Public Health interventions

## References

[1] Skivington K, Matthews L, Simpson SA, Craig P, Baird J, Blazeby JM, et al. A new framework for developing and evaluating complex interventions: update of Medical Research Council guidance. BMJ. 2021;374:n2061. 10.1136/bmj.n2061. 34593508 10.1136/bmj.n2061 PMC8482308

[2] Briggs A, Sculpher M, Claxton K. Decision modelling for health economic evaluation. Handbooks in health economic evaluation. Oxford, UK: Oxford University Press; 2006.

[3] NICE. Published Guidance, NICE advice and quality standards. https://www.nice.org.uk/guidance/published?ngt=Public%20health%20guidelines&ndt=Guidance (2023). Accessed.

[4] Brennan A, Chick SE, Davies R. A taxonomy of model structures for economic evaluation of health technologies. Health Econ. 2006;15(12):1295–310. 10.1002/hec.1148. 16941543 10.1002/hec.1148

[5] Boyd J, Wilson R, Elsenbroich C, Heppenstall A, Meier P. Agent-Based Modelling of Health Inequalities following the complexity turn in Public Health: a systematic review. Int J Environ Res Public Health. 2022;19(24): 16807. 10.3390/ijerph192416807. 36554687 10.3390/ijerph192416807 PMC9779847

[6] Emmert-Fees KMF, Karl FM, von Philipsborn P, Rehfuess EA, Laxy M. Simulation modeling for the economic evaluation of Population-based dietary policies: a systematic scoping review. Adv Nutr. 2021;12(5):1957–95. 10.1093/advances/nmab028. 33873201 10.1093/advances/nmab028 PMC8483966

[7] Wu G, Heppenstall A, Meier P, Purshouse R, Lomax N. A synthetic population dataset for estimating small area health and socio-economic outcomes in Great Britain. Sci Data. 2022;9(1):19. 10.1038/s41597-022-01124-9. 35058471 10.1038/s41597-022-01124-9 PMC8776798

[8] Breeze PR, Thomas C, Squires H, Brennan A, Greaves C, Diggle P, et al. Cost-effectiveness of population-based, community, workplace and individual policies for diabetes prevention in the UK. Diabet Med. 2017;34(8):1136–44. 10.1111/dme.13349. 28294392 10.1111/dme.13349 PMC5573930

[9] Virtanen SE, Galanti MR, Johansson PM, Feldman I. Economic evaluation of a brief counselling for smoking cessation in dentistry: a case study comparing two health economic models. BMJ Open. 2017;7(7): e016375. 10.1136/bmjopen-2017-016375. 28729321 10.1136/bmjopen-2017-016375 PMC5541608

[10] Willinger N, Steele J, Atkinson L, Liguori G, Jimenez A, Mann S, et al. Effectiveness of structured physical activity interventions through the evaluation of physical activity levels, adoption, Retention, maintenance, and Adherence Rates: a systematic review and Meta-analysis. J Phys Act Health. 2021;18(1):116–29. 10.1123/jpah.2019-0349. 33383569 10.1123/jpah.2019-0349

[11] Hubbard G, Gorely T, Ozakinci G, Polson R, Forbat L. A systematic review and narrative summary of family-based smoking cessation interventions to help adults quit smoking. BMC Fam Pract. 2016;17:17. 10.1186/s12875-016-0457-4. 27342987 10.1186/s12875-016-0457-4 PMC4921023

[12] Skinner R, Gonet V, Currie S, Hoddinott P, Dombrowski SU. A systematic review with meta-analyses of text message-delivered behaviour change interventions for weight loss and weight loss maintenance. Obes Rev. 2020;21(6). 10.1111/obr.12999. 10.1111/obr.12999 32043809

[13] Bates S, Bayley T, Norman P, Breeze P, Brennan A. A systematic review of methods to Predict Weight Trajectories in Health Economic Models of Behavioral Weight Management Programs: the potential role of psychosocial factors. Med Decis Making. 2020;40(1):90–105. 10.1177/0272989x19889897. 31789103 10.1177/0272989X19889897 PMC6985993

[14] Candio P, Pouwels KB, Meads D, Hill AJ, Bojke L, Williams C. Modelling decay in effectiveness for evaluation of behaviour change interventions: a tutorial for public health economists. Eur J Health Econ. 2022;23(7):1151–7. 10.1007/s10198-021-01417-7. 34914010 10.1007/s10198-021-01417-7 PMC9395462

[15] Carey RN, Connell LE, Johnston M, Rothman AJ, de Bruin M, Kelly MP, et al. Behavior change techniques and their mechanisms of action: a synthesis of Links described in published intervention literature. Ann Behav Med. 2019;53(8):693–707. 10.1093/abm/kay078. 30304386 10.1093/abm/kay078 PMC6636886

[16] Howlett N, Trivedi D, Troop NA, Chater AM. Are physical activity interventions for healthy inactive adults effective in promoting behavior change and maintenance, and which behavior change techniques are effective? A systematic review and meta-analysis. Transl Behav Med. 2019;9(1):147–57. 10.1093/tbm/iby010. 29506209 10.1093/tbm/iby010 PMC6305562

[17] Johnston M, Carey RN, Connell Bohlen LE, Johnston DW, Rothman AJ, de Bruin M, et al. Development of an online tool for linking behavior change techniques and mechanisms of action based on triangulation of findings from literature synthesis and expert consensus. Transl Behav Med. 2021;11(5):1049–65. 10.1093/tbm/ibaa050. 32749460 10.1093/tbm/ibaa050 PMC8158171

[18] Michie S, West R, Campbell R, Brown J, Gainforth H. ABC of Behaviour Change theories. London: Silverback Publishing; 2014.

[19] Prochaska JO, Velicer WF. The transtheoretical model of health behavior change. Am J Health Promot. 1997;12(1):38–48. 10.4278/0890-1171-12.1.38. 10170434 10.4278/0890-1171-12.1.38

[20] Ajzen I. The theory of planned behavior. Organ Behav Hum Decis Process. 1991;50(2):179–211.

[21] Bandura A. Social cognitive theory: an agentic perspective. Annu Rev Psychol. 2001;52:1–26. 10.1146/annurev.psych.52.1.1. 11148297 10.1146/annurev.psych.52.1.1

[22] Michie S, Carey RN, Johnston M, Rothman AJ, de Bruin M, Kelly MP, et al. From theory-inspired to theory-based interventions: a protocol for developing and testing a methodology for linking Behaviour Change techniques to theoretical mechanisms of action. Ann Behav Med. 2018;52(6):501–12. 10.1007/s12160-016-9816-6. 27401001 10.1007/s12160-016-9816-6 PMC6367898

[23] Prestwich A, Sniehotta FF, Whittington C, Dombrowski SU, Rogers L, Michie S. Does theory influence the effectiveness of health behavior interventions? Meta-analysis. Health Psychol. 2014;33(5):465–74. 10.1037/a0032853. 23730717 10.1037/a0032853

[24] Dalgetty R, Miller CB, Dombrowski SU. Examining the theory-effectiveness hypothesis: a systematic review of systematic reviews. Br J Health Psychol. 2019;24(2):334–56. 10.1111/bjhp.12356. 30793445 10.1111/bjhp.12356

[25] Sniehotta F, Presseau J, Araújo-Soares V. Time to retire the theory of planned behaviour. Health Psychol Rev. 2014;8(1):1–7. 25053004 10.1080/17437199.2013.869710

[26] West R. Time for a change: putting the Transtheoretical (stages of Change) Model to rest. Addiction. 2005;100(8):1036–9. 16042624 10.1111/j.1360-0443.2005.01139.x

[27] Michie S, van Stralen MM, West R. The behaviour change wheel: a new method for characterising and designing behaviour change interventions. Implement Sci. 2011;6: 42. 10.1186/1748-5908-6-42. 21513547 10.1186/1748-5908-6-42 PMC3096582

[28] Schluter M, Baeza A, Dresser G, Frank K, Goeneveld J, Jager W, et al. A framework for mapping and comparing behavioural theories in models of socio-ecological systems. Ecol Econ. 2017;131:21–35.

[29] Gigerenzer G, Gaissmaier W. Heuristic decision making. Ann Rev Psychol. 2011;62:451–82. 21126183 10.1146/annurev-psych-120709-145346

[30] Kahneman D. Thinking, fast and slow. London: Penguin Books Ltd; 2012.

[31] Thaler R, Sunstein C, Nudge. Improving decisions about health, wealth and happiness. New Haven: Yale University Press; 2008.

[32] Vlaev I, King D, Dolan P, Darzi A. The theory and practice of nudging: changing health behaviors. Public Adm Rev. 2016;76:550–61.

[33] Giddens A. Central problems in social theory: action, structure and contradiction in social analysis. Berkeley, US: University of California Press; 1979.

[34] Zhang S, Haye Kdl, Ji M, An R. Applications of social network analysis to obesity: a systematic review. Obes Etiology. 2018;19(7):976–88. 10.1111/obr.12684 29676508

[35] Hunter RF, de la Haye K, Murray JM, Badham J, Valente TW, Clarke M, et al. Social network interventions for health behaviours and outcomes: a systematic review and meta-analysis. PLoS Med. 2019;16(9): e1002890. 10.1371/journal.pmed.1002890. 31479454 10.1371/journal.pmed.1002890 PMC6719831

[36] Cascini F, Pantovic A, Al-Ajlouni YA, Failla G, Puleo V, Melnyk A, et al. Social media and attitudes towards a COVID-19 vaccination: a systematic review of the literature. EClinicalMedicine. 2022;48: 101454. 10.1016/j.eclinm.2022.101454. 35611343 10.1016/j.eclinm.2022.101454 PMC9120591

[37] Sterman J. Business dynamics: systems thinking and modeling for a Complex World. Singapore: McGraw-Hill; 2000.

[38] Squires H, Chilcott J, Akehurst R, Burr J, Kelly MP. A Framework for developing the Structure of Public Health Economic Models. Value Health. 2016;19(5):588–601. 10.1016/j.jval.2016.02.011. 27565276 10.1016/j.jval.2016.02.011

[39] Perkins HW, Berkowitz AD. Perceiving the community norms of alcohol use among students: some research implications for campus alcohol education programming. Int J Addict. 1986;21(9–10):961–76. 10.3109/10826088609077249. 3793315 10.3109/10826088609077249

[40] Jetten J, Haslam C, Haslam SA. The social cure: identity, health and well-being. London: Psychology Press; 2012.

[41] Holt-Lunstad J, Smith TB, Layton JB. Social relationships and mortality risk: a meta-analytic review. PLoS Med. 2010;7(7): e1000316. 10.1371/journal.pmed.1000316. 20668659 10.1371/journal.pmed.1000316 PMC2910600

[42] Caldwell AE, Masters KS, Peters JC, Bryan AD, Grigsby J, Hooker SA, et al. Harnessing centred identity transformation to reduce executive function burden for maintenance of health behaviour change: the maintain IT model. Health Psychol Rev. 2018;12(3):231–53. 10.1080/17437199.2018.1437551. 29402182 10.1080/17437199.2018.1437551 PMC6124500

[43] Kelly MP, Stewart E, Morgan A, Killoran A, Fischer A, Threlfall A, et al. A conceptual framework for public health: NICE’s emerging approach. Public Health. 2009;123(1):e14–20. 10.1016/j.puhe.2008.10.031. 19100588 10.1016/j.puhe.2008.10.031

[44] Public Health England. Health matters: Prevention - a life course approach. 2019. https://www.gov.uk/government/publications/health-matters-life-course-approach-to-prevention. Accessed Sept 2024.

[45] Glanz K, Rimer BK, Viswanath K. Chaper 3: Ecological models of health behavior. In: K. G, Rimer BK, Viswanath K, editors. Health behavior: theory, research and practice. 5th ed. San Francisco: Wiley; 2015.

[46] Dahlgren G, Whitehead M. Policies and strategies to promote social equity in health. Sweden: Institute for Future Studies. Stockholm; 1991.

[47] McManus J, Constable M, Bunten A, Chadborn T. Improving people’s health: applying behavioural and social sciences to improve population health and wellbeing in England. Public Health England; 2018. https://www.gov.uk/government/publications/improving-peoples-health-applying-behavioural-and-social-sciences. Accessed Sept 2024.

[48] Greenhalgh T, Robert G, Macfarlane F, Bate P, Kyriakidou O. Diffusion of innovations in service organizations: systematic review and recommendations. Milbank Q. 2004;82(4):581–629. 10.1111/j.0887-378X.2004.00325.x. 15595944 10.1111/j.0887-378X.2004.00325.x PMC2690184

[49] Bardach AE, Alcaraz AO, Ciapponi A, Garay OU, Riviere AP, Palacios A, et al. Alcohol consumption’s attributable disease burden and cost-effectiveness of targeted public health interventions: a systematic review of mathematical models. BMC Public Health. 2019;19(1):1378. 10.1186/s12889-019-7771-4. 31655600 10.1186/s12889-019-7771-4 PMC6815367

[50] Leao T, Kunst AE, Perelman J. Cost-effectiveness of tobacco control policies and programmes targeting adolescents: a systematic review. Eur J Public Health. 2018;28(1):39–43. 10.1093/eurpub/ckx215. 29267928 10.1093/eurpub/ckx215 PMC5881796

[51] Zanganeh M, Adab P, Li B, Frew E. A systematic review of methods, Study Quality, and results of economic evaluation for Childhood and adolescent obesity intervention. Int J Environ Res Public Health. 2019;16(3): 485. 10.3390/ijerph16030485. 30743995 10.3390/ijerph16030485 PMC6388206

[52] Zhou X, Siegel KR, Ng BP, Jawanda S, Proia KK, Zhang X, et al. Cost-effectiveness of diabetes Prevention interventions Targeting High-risk individuals and whole populations: a systematic review. Diabetes Care. 2020;43(7):1593–616. 10.2337/dci20-0018. 33534726 10.2337/dci20-0018

[53] Kwon J, Squires H, Franklin M, Lee Y, Young T. Economic models of community-based falls prevention: a systematic review with subsequent commissioning and methodological recommendations. BMC Health Serv Res. 2022;22(1):316. 10.1186/s12913-022-07647-6. 35255898 10.1186/s12913-022-07647-6 PMC8902781

[54] Squires H, Kelly MP, Gilbert N, Sniehotta F, Purshouse RC, Garcia L, et al. Incorporating the influences on behaviour within public health economic models (PHEM-B). https://sites.google.com/sheffield.ac.uk/behaviourinmodels/home. Accessed June 2024. 10.1186/s12889-024-20225-1 PMC11475213 39395958

[55] Squires H, Kelly MP, Gilbert N, Sniehotta F, Purshouse RC. The long-term effectiveness and cost-effectiveness of public health interventions; how can we model behavior? A review. Health Econ. 2023. 10.1002/hec.4754. 37681282 10.1002/hec.4754 PMC10843043

[56] Probst C, Vu TM, Epstein J, Nielsen E, Buckley C, Brennan A, et al. The normative underpinnings of population-level alcohol use: an individual-level simulation model. Health Educ Behav. 2020;47(2):224–34. 32090651 10.1177/1090198119880545 PMC7069782

[57] Meier P, Purshouse R, Bain M, Bambra C, Bentall R, Birkin M, et al. The SIPHER Consortium: introducing the new UK hub for systems science in public health and health economic research. Wellcome Open Res. 2019;4:174. 10.12688/wellcomeopenres.15534.1. 31815191 10.12688/wellcomeopenres.15534.1 PMC6880277

[58] Bates SE, Thomas C, Islam N, Ahern AL, Breeze P, Griffin S, et al. Using health economic modelling to inform the design and development of an intervention: estimating the justifiable cost of weight loss maintenance in the UK. BMC Public Health. 2022;22(1):290. 10.1186/s12889-022-12737-5. 35151300 10.1186/s12889-022-12737-5 PMC8840781

[59] West R, Michie S, Atkins L, Chadwick P, Lorencatto F. Achieving behaviour change: a guide for local government and partners. Public Health England; 2019. https://www.gov.uk/government/publications/behaviour-change-guide-for-local-government-and-partners. Accessed Sept 2024.

[60] Kwasnicka D, Dombowski SW, Sniehotta M. Theoretical explanations for maintenance of behaviour change: a systematic review of behavioural theories. Health Psychol Rev. 2016;10(3):277–96. 26854092 10.1080/17437199.2016.1151372 PMC4975085

[61] Madigan CD, Fong M, Howick J, Kettle V, Rouse P, Hamilton L, et al. Effectiveness of interventions to maintain physical activity behavior (device-measured): systematic review and meta-analysis of randomized controlled trials. Obes Rev. 2021;22(10). 10.1111/obr.13304. 10.1111/obr.13304 34129276

[62] Michie S, West R, Finnerty AN, Norris E, Wright AJ, Marques MM, et al. Representation of behaviour change interventions and their evaluation: development of the Upper Level of the Behaviour Change intervention ontology. Wellcome Open Res. 2020;5:123. 10.12688/wellcomeopenres.15902.2. 33614976 10.12688/wellcomeopenres.15902.1 PMC7868854

[63] Project HBC. Behaviour Change Intervention Ontology. https://www.bciontology.org/. Accessed Sept 2023.

[64] Hale J, Jofeh C, Chadwick P. Decarbonising existing homes in Wales: a participatory behavioural systems mapping approach. UCL Open Environ. 2022;4. 10.14324/111.444/ucloe.000047. 10.14324/111.444/ucloe.000047 PMC10208331 37228458

[65] Craven LK. System effects: a hybrid methodology for exploring the determinants of Food In/Security. Annals Am Association Geographers. 2017;107(5):1011–27.

[66] Allender S, Owen B, Kuhlberg J, Lowe J, Nagorcka-Smith P, Whelan J, et al. A community based systems Diagram of obesity causes. PLoS ONE. 2015;10(7): e0129683. 10.1371/journal.pone.0129683. 26153893 10.1371/journal.pone.0129683 PMC4496094

[67] Gilbert N. Agent-based models. Quantitative applications in the social sciences. London: SAGE Publications Inc; 2020.

[68] Giabbanelli PJ, Crutzen R. Using agent-based models to develop public policy about food behaviours: future directions and recommendations. Comput Math Methods Med. 2017;2017. 10.1155/2017/5742629. 10.1155/2017/5742629 PMC5379081 28421127

[69] Vu TM, Probst C, Nielsen A, Bai H, Buckley C, Meier P, Strong M, Brennan A, Purshouse R. A Software Architecture for mechanism-based Social systems Modelling in Agent-based Simulation models. J Artif Soc Social Simulation. 2020;23(3):1. 10.18564/jasss.4282 PMC7743915 33335448

[70] Occhipinti JA, Skinner A, Iorfino F, Lawson K, Sturgess J, Burgess W, et al. Reducing youth suicide: systems modelling and simulation to guide targeted investments across the determinants. BMC Med. 2021;19(1):61. 10.1186/s12916-021-01935-4. 33706764 10.1186/s12916-021-01935-4 PMC7952221

[71] Breeze PR, Squires H, Ennis K, Meier P, Hayes K, Lomax N, et al. Guidance on the use of complex systems models for economic evaluations of public health interventions. Health Econ. 2023. 10.1002/hec.4681. 37081811 10.1002/hec.4681 PMC10947434

[72] Knoke D, Yang S. Social Network Analysis: Third Edition. Applications in the Social Sciences. California, US: SAGE; 2020.

[73] Badham J, McAneney H, Dunne L, Kee F, Thurston A, Hunter RF. The importance of social environment in preventing smoking: an analysis of the Dead cool intervention. BMC Public Health. 2019;19(1):1182. 10.1186/s12889-019-7485-7. 31462249 10.1186/s12889-019-7485-7 PMC6714405

[74] Smith J, Burow J. Using Eco Network Data To Inform Agent-based models of Diffusion. Sociol Methods Res. 2020;49(4):1018–63.

[75] Snijders TAB. Stochastic actor-oriented models for Network Dynamics. Annual Rev Stat Its Application. 2017;4:343–63.

[76] Crooks A, Malleson N, Manley E, Heppenstall A. Agent-based modelling & geographical information systems. A practical primer. London: SAGE Publications Ltd; 2019.

[77] Crols T, Malleson N. Quantifying the ambient population using hourly population footfall data and an agent-based model of daily mobility. Geoinformatica. 2019;23(2):201–20. 10.1007/s10707-019-00346-1. 32647494 10.1007/s10707-019-00346-1 PMC7328437

[78] Robinson S. Simulation: The practice of Model Development and Use. London, UK: Red Globe; 2014.

[79] Karnon J, Stahl J, Brennan A, Caro JJ, Mar J, Moller J, et al. Modeling using discrete event simulation: a report of the ISPOR-SMDM modeling Good Research practices Task Force–4. Value Health. 2012;15(6):821–7. 10.1016/j.jval.2012.04.013. 22999131 10.1016/j.jval.2012.04.013

[80] Brailsford S, Harper PR, Sykes J. Incorporating human behaviour in simulation models of screening for breast cancer. Eur J Oper Res. 2012;219:491–507.

[81] Sullivan W. Exploring the importance of links between health behaviours for economic evaluations of behaviour-change strategies: a case study considering the link between smoking cigarettes and drinking alcohol. White Rose Online: University of Sheffield; 2014.

[82] Bianconcini S, Bollen KA. The latent variable-autoregressive latent trajectory model: a general framework for longitudinal data analysis. Struct Equ Modeling. 2018;25(5):791–808. 10.1080/10705511.2018.1426467. 31293345 10.1080/10705511.2018.1426467 PMC6619429

[83] Bates S. Incorporating psychological mechanisms of action in a health economic model of obesity. 2021.

[84] Bell A. Age period cohort analysis: a review of what we should and shouldn’t do. Ann Hum Biol. 2020;47(2):208–17. 10.1080/03014460.2019.1707872. 32429768 10.1080/03014460.2019.1707872

[85] O’Hagan A. Expert Knowledge Elicitation: subjective but scientific. Am Stat. 2019;73:69–81.

[86] Bojke L, Soares M, Claxton K, Colson A, Fox A, Jackson C, et al. Developing a reference protocol for structured expert elicitation in health-care decision-making: a mixed-methods study. Health Technol Assess. 2021;25(37):1–124. 10.3310/hta25370. 34105510 10.3310/hta25370 PMC8215568

[87] Tubaro P, Casilli A. An ethnographic seduction: how qualitative research and agent-based models can benefit each other. Bull Sociol Methodol. 2010;106(1):59–74.

[88] Beach D, Pedersen R. Process-tracing methods: foundations and guidelines. University of Michigan Press; 2019.

[89] Flemming K, Booth A, Garside R, Tuncalp O, Noyes J. Qualitative evidence synthesis for complex interventions and guideline development: clarification of the purpose, designs and relevant methods. BMJ Glob Health. 2019;4(Suppl 1): e000882. 10.1136/bmjgh-2018-000882. 30775015 10.1136/bmjgh-2018-000882 PMC6350756

[90] Michie S, Atkins L, West R. The behaviour change wheel. A guide to designing interventions. London: Silverback Publishing; 2014.

[91] Brierley ML, Smith LR, Bailey DP, Ojo SO, Hewson DJ, Every SA, et al. Evaluating a multi-component intervention to reduce and break up office workers’ sitting with sit-stand desks using the APEASE criteria. BMC Public Health. 2022;22(1):458. 10.1186/s12889-022-12794-w. 35255850 10.1186/s12889-022-12794-w PMC8902706

[92] Goldschmidt S, Schmidt ME, Steindorf K. Maintenance of physical activity after exercise interventions: a systematic review and meta-analysis. Ann Oncol. 2022;33:S226–S. 10.1016/j.annonc.2022.03.238.

[93] van der Heijden LB, Feskens EJM, Janse AJ. Maintenance interventions for overweight or obesity in children: a systematic review and meta-analysis. Obes Rev. 2018;19(6):798–809. 10.1111/obr.12664. 29363283 10.1111/obr.12664

[94] Flore G, Preti A, Carta MG, Deledda A, Fosci M, Nardi AE, et al. Weight Maintenance after Dietary Weight loss: systematic review and Meta-analysis on the effectiveness of behavioural intensive intervention. Nutrients. 2022;14(6): 1259. 10.3390/nu14061259. 35334917 10.3390/nu14061259 PMC8953094

[95] Murray JM, Brennan SF, French DP, Patterson CC, Kee F, Hunter RF. Effectiveness of physical activity interventions in achieving behaviour change maintenance in young and middle aged adults: a systematic review and meta-analysis. Soc Sci Med. 2017;192:125–33. 10.1016/j.socscimed.2017.09.021. 28965003 10.1016/j.socscimed.2017.09.021

[96] Hoffmann TC, Glasziou PP, Boutron I, Milne R, Perera R, Moher D, et al. Better reporting of interventions: template for intervention description and replication (TIDieR) checklist and guide. BMJ. 2014;348: g1687. 10.1136/bmj.g1687. 24609605 10.1136/bmj.g1687

[97] PHE. Health economics: a guide for public health teams. https://www.gov.uk/guidance/health-economics-a-guide-for-public-health-teams#the-cost-effectiveness-of-specific-topic-areas (2017). Accessed.

[98] Grimm V, Railsback SF, Vincenot CE, Berger U, Gallagher C, DeAngelis DL, et al. The ODD Protocol for describing Agent-based and other Simulation models: a second update to improve clarity, replication, and structural realism. J Artif Soc Social Simulation: JASSS. 2020;23(2):7.33204215

[99] Boyd J, Sexton O, Angus C, Meier P, Purshouse RC, Holmes J. Causal mechanisms proposed for the alcohol harm paradox-a systematic review. Addiction. 2022;117(1):33–56. 10.1111/add.15567. 33999487 10.1111/add.15567 PMC8595457

[100] Garcia LMT, Roux AVD, Martins ACR, Yang Y, Florindo AA. Development of a dynamic framework to explain population patterns of leisure-time physical activity through agent-based modeling. Int J Behav Nutr Phys Activity. 2017;14:14. 10.1186/s12966-017-0553-4. 10.1186/s12966-017-0553-4 PMC5568398 28830527

[101] Ernecoff NC, Keane CR, Albert SM. Health behavior change in advance care planning: an agent-based model. BMC Public Health. 2016;16: 193. 10.1186/s12889-016-2872-9. 26924203 10.1186/s12889-016-2872-9 PMC4770523

[102] Buckley C, Field M, Vu TM, Brennan A, Greenfield TK, Meier PS, et al. An integrated dual process simulation model of alcohol use behaviours in individuals, with application to US population-level consumption, 1984–2012. Addict Behav. 2022;124: 107094. 10.1016/j.addbeh.2021.107094. 34530207 10.1016/j.addbeh.2021.107094 PMC8529781

[103] Michie S, West R. Sustained behavior change is key to preventing and tackling future pandemics. Nat Med. 2021;27(5):749–52. 10.1038/s41591-021-01345-2. 33972794 10.1038/s41591-021-01345-2

[104] Choi YJ, Ailshire JA, Crimmins EM. Living alone, social networks in neighbourhoods, and daily fruit and vegetable consumption among middle-aged and older adults in the USA. Public Health Nutr. 2020;23(18):3315–23. 10.1017/S1368980020002475. 32792025 10.1017/S1368980020002475 PMC7736134

[105] Storey KE, Stearns JA, McLeod N, Montemurro G. A social network analysis of interactions about physical activity and nutrition among APPLE schools staff. SSM Popul Health. 2021;14: 100763. 10.1016/j.ssmph.2021.100763. 33748390 10.1016/j.ssmph.2021.100763 PMC7966860

[106] Luke DA, Hammond RA, Combs T, Sorg A, Kasman M, Mack-Crane A, et al. Tobacco Town: computational modeling of Policy options to reduce Tobacco Retailer Density. Am J Public Health. 2017;107(5):740–6. 10.2105/AJPH.2017.303685. 28398792 10.2105/AJPH.2017.303685 PMC5388950

[107] Bicket M, Christie I, Gilbert N, Hills D, Penn A, Wilkinson H. Magenta book 2020 supplementary guide: handling complexity in policy evaluation. HM Treasury; 2020. https://www.gov.uk/government/publications/the-magenta-book. Accessed Sept 2024.

[108] Claxton K, Sculpher M, McCabe C, Briggs A, Akehurst R, Buxton M, et al. Probabilistic sensitivity analysis for NICE technology assessment: not an optional extra. Health Econ. 2005;14(4):339–47. 10.1002/hec.985. 15736142 10.1002/hec.985

[109] Rothery C, Strong M, Koffijberg HE, Basu A, Ghabri S, Knies S, et al. Value of information analytical methods: Report 2 of the ISPOR value of information analysis emerging good practices task force. Value Health. 2020;23(3):277–86. 10.1016/j.jval.2020.01.004. 32197720 10.1016/j.jval.2020.01.004 PMC7373630

[110] Vu TM, Buckley C, Bai H, Nielsen A, Probst C, Brennan A et al. Multiobjective genetic programming can improve the explanatory capabilities of mechanism-based models of social systems. Complexity. 2020:1–19. 10.1155/2020/8923197. 10.1155/2020/8923197 PMC7743914 33335382

[111] Pouwels X, Sampson CJ, Arnold RJG, Open Source Models Special Interest G. Opportunities and barriers to the Development and Use of Open Source Health Economic models: a Survey. Value Health. 2022;25(4):473–9. 10.1016/j.jval.2021.10.001. 35365297 10.1016/j.jval.2021.10.001

[112] Sampson CJ, Arnold R, Bryan S, Clarke P, Ekins S, Hatswell A, et al. Transparency in decision modelling: What, Why, Who and How? Pharmacoeconomics. 2019;37(11):1355–69. 10.1007/s40273-019-00819-z. 31240636 10.1007/s40273-019-00819-z PMC8237575

