# Supplementary material for: The PHEM-B toolbox of methods for incorporating the influences on Behaviour into Public Health Economic Models
Source: BMC Public Health. 2024 Oct 12;24:2794. doi: 10.1186/s12889-024-20225-1 (PMC11475213; doi:10.1186/s12889-024-20225-1)
Supplement: Supplementary file 1 — Supplementary Material 1. [file 12889_2024_20225_MOESM1_ESM.docx]

1. **Glossary**

| **Term** | **Description** |
| --- | --- |
| Behaviour change technique | An observable, replicable and irreducible component of an intervention designed to alter or redirect causal processes that regulate behaviour. |
| Behavioural science | A set of disciplines to understand how and why people behave the way in which they do. In this paper, we focus on psychology, sociology and behavioural economics. |
| Calibration | An iterative process to estimate model parameters so that model outputs match observational outcomes, where there may be no data on the model parameters or they are unobservable. |
| Cohort model | A model that simulates a population as aggregated groups with population average values. |
| Health economic modelling | The synthesis and comparison of costs and benefits of alternative interventions, usually over an individual’s lifetime to capture all impacts of the interventions to inform policy decisions about how to best spend limited resources. |
| Incidence | The occurrence of new cases of disease in the population over a specific time period. |
| Individual level model | A model that simulates individuals as single elements which allows the characteristics and heterogeneity of the population to be reflected. |
| Longitudinal data | Data that records the same information for individuals from the same population at multiple time points. |
| Place | Physical spaces where behaviours can occur, which includes both the built (e.g. public house) and natural environment (e.g. green spaces). |
| Place in the social network | The number of social connections an individual has and the influence they may have within the network |
| Prevalence | The proportion of people within a population who have a disease at a specific point in time. |
| Price elasticity | The percentage change in quantity demanded (or supplied) divided by the percentage change in price. |
| Public health intervention | Actions or activities that aim to make a person or population behave differently from how they would otherwise have done. |
| Social science | A set of disciplines which study the behaviours and interactions of individuals, communities and societies. |
| Systems map | A visual representation of the system showing relationships between factors using arrows. |
| Validation | How accurately a model reflects outcomes within the real world. |

1. **A COM-B analysis of the use of a toolbox by modellers to incorporate the influences of behaviour into health economic models**

| COM-B elements | What needs to change | Intervention types required |
| --- | --- | --- |
| Physical capability | The accessibility of the toolbox to health economic modellers who may be visually impaired. | Enablement by providing an accessible version of the toolbox. |
| Psychological capability | The knowledge, skills and self-regulation to be able to incorporate these approaches within health economic models | Education and Training to develop the knowledge and skills needed to incorporate these approaches within models, including demonstrating within case studies. |
| Reflective motivation | The desire to use the toolbox | Education to provide the rationale for why incorporating these methods within health economic models can be important. |
| Automatic motivation | The automatic use of existing guidance for assessing public health interventions within models | Persuasion to show the potentially negative impacts of developing a model without considering these approaches. |
| Physical opportunity | The methods, data and resources to be able to incorporate behavioural theory into models | Enablement by developing methods and resources so that modellers can incorporate behavioural theories within models |
| Social opportunity | To become the norm as part of the modelling process | Modelling (i.e., providing an example for people to aspire to or imitate) by sharing case studies and making code open access. |
|  | The opportunity to involve behavioural scientists in projects | Environmental restructuring by building networks between modellers and behavioural/ social scientists. |

1. **The School for Public Health Research (SPHR) diabetes prevention model**

The SPHR diabetes prevention model is an individual level microsimulation assessing diabetes prevention interventions at the individual, community and population level [1]. It simulates a population with sociodemographic variables (e.g., age, sex, ethnicity, deprivation) and clinical variables (e.g., body mass index (BMI), cholesterol, blood pressure, blood glucose). Statistical analyses link change in BMI to changes in blood pressure, cholesterol and blood glucose, conditional on individual sociodemographic and family history variables. The level of BMI then affects the probability of an individual having a range of diseases (diabetes, CVD, osteoarthritis) and health and cost outcomes. Risk factors and clinical events are updated for each individual annually. There are no interactions between individuals or with their environment. Within the original version of the model, behaviour was not incorporated, only the impacts of physical activity or dietary interventions upon clinical risk factors.

A key purpose of the SPHR diabetes prevention model was to be able to compare a wide range of diabetes prevention interventions, from individual level to community to population level. Previous models had not incorporated multiple risk factors for diabetes and its related complications and co-morbidities, and as such they had just focused upon specific intervention types. Effectiveness evidence used within the model was based upon one study for each intervention due to the large heterogeneity between studies of similar interventions. Interventions included were soft drinks taxation (outcome was change in BMI conditional on age), workplace health promotion (outcome was percentage of individuals who made a healthy change in terms of fruit consumption or reduced fat milk, but the study did not report the magnitude of the effect), opening a large supermarket in an area of deprivation to improve access to fruit and vegetables (outcome was reduced blood pressure), dietary advice in high risk communities (outcomes were effects on HbA1c and systolic blood pressure, independently of the effects on BMI at 6 and 12 months) and a diabetes prevention programme to identify and provide lifestyle advice to high risk individuals (outcomes were BMI, HbA1c, systolic blood pressure and total cholesterol at 12 months). Intervention study results are not generally separated by intervention period and maintenance period. Since the model did not include physical activity, local transport policy was excluded from the evaluation. Thus, the exclusion of behaviour from the model limited what could be assessed.

Within the published paper of the model, there is a great level of detail about the model methodology [1]. However, for extrapolation of the intervention effects, it is reported that ‘Data were not available for the maintenance of metabolic changes for each intervention. The effectiveness decreased linearly after the first year, reaching zero effect after 5 years, in line with observations from studies of dietary counselling for weight loss [2].’ Dansinger et al. [2] meta-analysed studies greater than 16 weeks duration, reporting the effect of dietary counselling for weight loss. This study suggests that dietary counselling interventions lead to modest weight loss on average, that gradually increases during the intervention maintenance period which is reported up to 5 years. Dansinger et al. [2] report that the interventions, study samples and weight changes are heterogeneous, and each intervention is associated with a different slope for the weight increase in the maintenance period. However, within the diabetes prevention model the same slope was used for all interventions.

**3.1 Applying the PHEM-B toolbox to the SPHR diabetes prevention model**

***1) Collaboration between health economic modellers and behavioural/ social scientists***

During the diabetes prevention project there was substantial input from a range of clinical experts, but no collaboration or consultation with behavioural scientists. Behaviour and its influences were not considered explicitly. Specific behaviours, for example intake of fruit and vegetables, were included if they happened to be outputs of an intervention study where the intervention was effective. Behavioural/ social scientists could have provided input around which behaviours might be most important regarding preventing diabetes, potential interactions between behaviours, important influences on the dietary and physical activity behaviours, the theories associated with the development of the interventions, and alternative extrapolation assumptions about the different interventions [3].

Within the diabetes prevention project, interventions were chosen by drawing upon literature reviews of intervention effectiveness studies and consulting with stakeholders to determine which of the effective interventions to focus upon within the model. This could be thought of as one iteration of a cycle of intervention evaluation and development. The findings of the model suggested that the diabetes prevention programme was by far the most cost-effective option, although it may increase health inequalities. The soft drinks tax and access to fruit and vegetables were the next most cost-effective, and they were also predicted to reduce health inequalities. The community dietary advice intervention had poor uptake, which may be the reason it was not considered to be value for money. In attempting to model these interventions, studies generally had a maximum of 12-months follow up and inconsistent outcomes were reported. It would have been useful to have had longer follow up, with a range of outcomes reported including the impact of the interventions upon metabolic trajectories, behaviours, and the influences of behaviour. In many cases these outcomes were reported on an aggregate basis for the whole population of interest. Instead, it would be beneficial to report uptake and outcomes either at the individual level or in terms of key subgroups of interest. These findings from the modelling could be discussed with behavioural scientists who are developing new diabetes prevention interventions to help inform data collection. Pre-trial evaluation of potential interventions could also be tested within the model to assess whether they are likely to be cost-effective prior to undertaking an intervention effectiveness study.

***2) Reviewing the literature for theories used to develop the interventions and behaviour maintenance studies***

For each intervention assessed, the study utilised within the model was reviewed to identify any theory upon which the interventions were based.

Soft drinks tax: This was an econometric study, based upon the relationship between price and consumption.

Workplace health promotion: This intervention was about providing healthy meals and was developed previously in other settings and then applied to the workplace within this study. It is unclear whether it was based upon any theory originally.

New large supermarket in deprived area to increase access to fruit and vegetables: This intervention was part of a big government initiative to tackle ‘food deserts’ where socially deprived individuals did not live within a close distance to food-retail provision. This was a before and after study and the analysis of the intervention was testing the theory that proximity to food-retail provision would impact nutritional quality of the diet of individuals with a low income.

Cookery classes & dietary advice in high-risk communities: This intervention involved cookery classes and information on a Mediterranean-type diet, healthy eating and healthy recipes given to 75 female patients with rheumatoid arthritis living in socially deprived areas of Glasgow. No theory is discussed in the paper. Mediterranean diets have been associated with increased survival and the prevention of coronary heart disease within other studies. The intervention group was not randomised, and instead determined by availability of the individuals to attend the cookery classes close to their home.

Diabetes prevention programme (DPP): This was a set of behaviour change techniques which could be used pragmatically. The effectiveness of the DPP was based upon a meta-analysis of many heterogeneous studies.

None of the included intervention studies presented theory of change or logic models. There is therefore limited formal theory to inform the behavioural systems map based upon these studies. However, many of these studies are not recent, and the use of theory in intervention development is increasing.

A search for behaviour maintenance studies was not undertaken during the project. A search in Web of Science has been undertaken using the following search terms to assess what evidence was available to Jan 2023: main* (title) AND behavi* (title) AND intervention* (topic) AND post* (topic)

Any studies which reported results 3 months+ following the intervention relating to physical activity or diet in adults were included. This resulted in 18 relevant studies, including five systematic reviews [4-8]. Whilst there is reasonable evidence about behaviour maintenance at 3 months post intervention, there is a dearth of evidence beyond 2 years. Studies do, however, suggest which elements of interventions are associated with behaviour (physical activity) maintenance which may be informative in making long-term assumptions about the interventions. For example, Howlett et al. [6] reported that the behaviour change techniques ‘action planning’, ‘instruction on how to perform the behaviour’, ‘prompts/ cues’, ‘behaviour practice/ rehearsal’, ‘graded tasks’ and ‘self-reward’ were effective at least 6 months postintervention. Thus, interventions including these behaviour-change techniques, such as the diabetes prevention programme, may be more likely to maintain behaviour than other interventions that do not.

***3) Apply the behaviour change intervention ontology [9] to describe interventions to model***

The below table uses the behaviour change techniques from the behaviour change intervention ontology to be clearer about the content of each intervention.

| **Intervention as described in paper** | **Behaviour change technique** |
| --- | --- |
| Soft drinks tax | Restructure the social environment BCT  [BCIO:050349](https://www.bciosearch.org/BCIO:050349) |
| Workplace health promotion | Inform about health consequences BCT  [BCIO:007063](https://www.bciosearch.org/BCIO:007063)  Restructure the physical environment BCT  [BCIO:050348](https://www.bciosearch.org/BCIO:050348) |
| New large supermarket in deprived area to increase access to fruit and vegetables | Restructure the physical environment BCT  [BCIO:050348](https://www.bciosearch.org/BCIO:050348) |
| Dietary advice and cookery classes in high risk communities | Instruct how to perform a behaviour BCT  [BCIO:007058](https://www.bciosearch.org/BCIO:007058)  Inform about health consequences BCT  [BCIO:007063](https://www.bciosearch.org/BCIO:007063)  Demonstrate the behaviour BCT  [BCIO:007055](https://www.bciosearch.org/BCIO:007055)  Prompt mental rehearsal of successful performance BCT  [BCIO:007138](https://www.bciosearch.org/BCIO:007138) |
| Diabetes prevention programme | Could be any of the following, used pragmatically:  Goal setting BCT  [BCIO:007002](https://www.bciosearch.org/BCIO:007002)  Action planning BCT  [BCIO:007010](https://www.bciosearch.org/BCIO:007010)  Review outcome goal BCT  [BCIO:007013](https://www.bciosearch.org/BCIO:007013)  Provide feedback on behaviour BCT  [BCIO:007023](https://www.bciosearch.org/BCIO:007023)  Self-monitor behaviour BCT  [BCIO:007024](https://www.bciosearch.org/BCIO:007024)  Social support BCT  [BCIO:007028](https://www.bciosearch.org/BCIO:007028)  Inform about health consequences BCT  [BCIO:007063](https://www.bciosearch.org/BCIO:007063)  Informational mode of delivery  [BCIO:011001](https://www.bciosearch.org/BCIO:011001)  Belief about the credibility of a message's source  [BCIO:006142](https://www.bciosearch.org/BCIO:006142)  Consider pros and cons BCT  [BCIO:007069](https://www.bciosearch.org/BCIO:007069)  Prompt comparative imagining of future outcomes BCT  [BCIO:007070](https://www.bciosearch.org/BCIO:007070)  Persuade about personal capability BCT  [BCIO:007137](https://www.bciosearch.org/BCIO:007137) |

The content of each intervention is now much clearer to all stakeholders and consistent terminology can be used for intervention development, delivery, evaluation, replication and fidelity checking. Using this ontology, it is easier to understand which studies may (or may not) be appropriately included within any meta-analysis of the effectiveness of interventions. This information can also be used to help develop the behavioural map.

The diabetes model was sufficiently flexible to allow the assessment of the impact of a combination of interventions. The effect of the interventions could be combined additively, synergistically (greater than additive), or antagonistically (multiple interventions are less effective than their additive effect), though there was no clear decision reported about which was most appropriate or why. The content of each intervention in terms of the behaviour change taxonomy and how they influence behaviour can be useful for choosing which of the above is the most appropriate. For example, workplace health promotion and cookery classes and dietary advice in high-risk communities both include information about health consequences, but the rest of the intervention content is quite different. Since they are also targeted at different locations, an additive effect would likely be most appropriate for these two interventions. Similarly, there is very little overlap in the content between cookery classes and dietary advice in high-risk communities and the soft drinks tax, which means that they may complement each other. This suggests that either an additive or synergistic relationship would be most appropriate for these two interventions.

***4) Behavioural system mapping***

A behavioural system map could be produced in consultation/ collaboration with behavioural scientists. In practice the maps would be developed iteratively based upon evidence, drawing upon relevant theories (see point (3)) and alongside behavioural scientists and other stakeholders. Figures 1 and 2 show two illustrative maps developed in a freely available software package called Participatory System Mapper (PRSM) [10]. First, key behaviours of interest are identified (shown in the blue boxes). Figure 1 draws on the COM-B model of behaviour to consider the influences on the behaviours, with capabilities (pink boxes), opportunities (green boxes) and motivations (orange boxes) linked to the behaviours (blue boxes). The interventions (purple ovals) are then incorporated.

This illustrative map suggests that none of the interventions considered here will change all three of capability, opportunity and motivation, thus the combination of interventions may be important. If all of capability, opportunity and motivation are addressed by the interventions, then it may be reasonable to assume that the intervention would have an effect for longer than if only one or two of these are addressed.

Figure 1: Illustrative behavioural map for dietary behaviours using COM-B


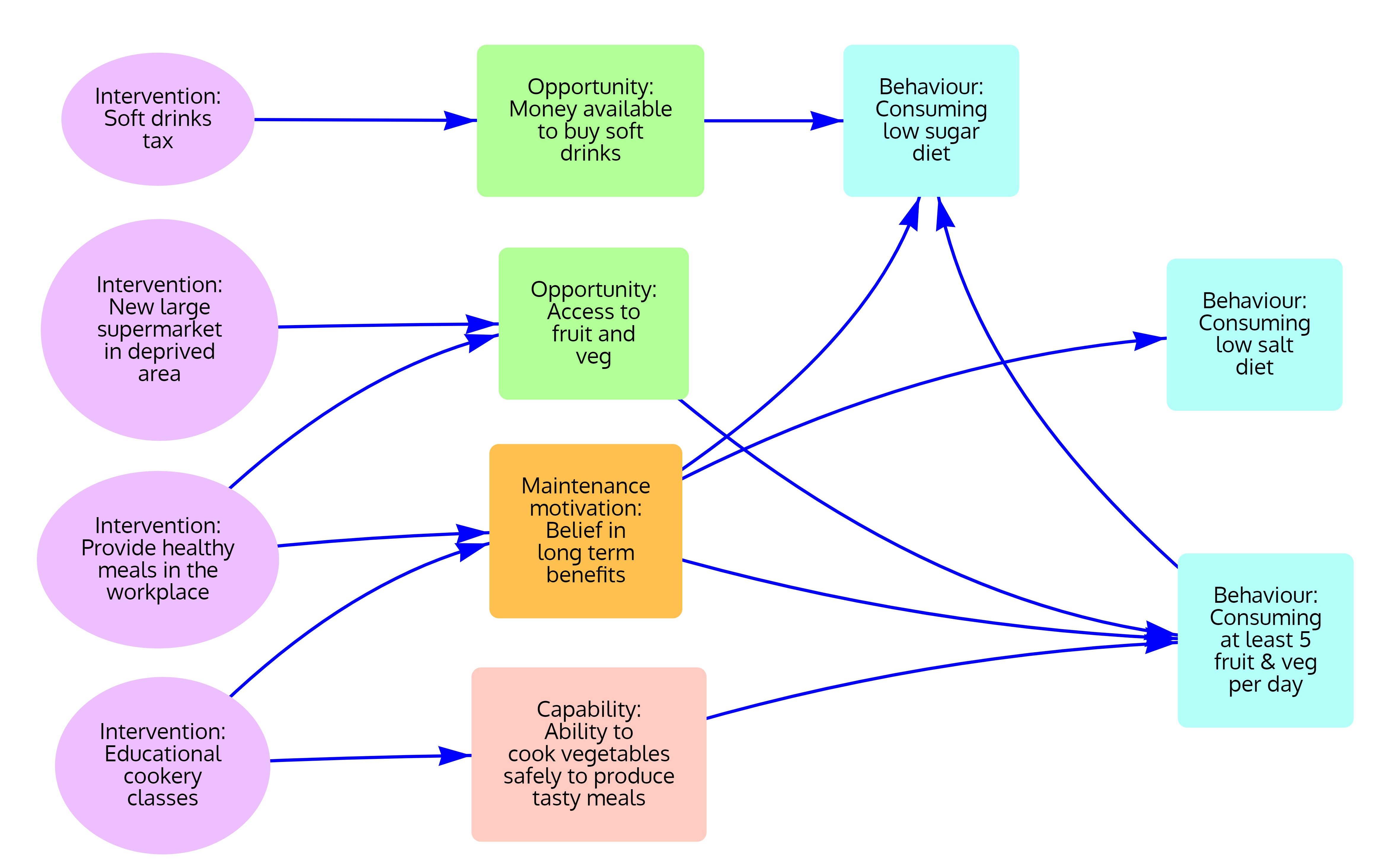


Alternatively, the behavioural map could be based on behaviour maintenance themes. An illustrative example of this is shown in Figure 2. The key additions to this map are habits and self-regulation and there are feedback loops between these and behaviour.

If these variables were collected within the intervention studies at multiple time points, then it may be possible to quantify these relationships, however in the absence of such data, these maps can be used to understand how the interventions are impacting upon behaviour and how likely their effects will be maintained. For example, any benefit from providing only a new supermarket in a deprived area is unlikely to be maintained because the intervention does not provide individuals with the motivation and resources to buy healthy foods. However, if this was combined with other interventions which increase maintenance motivation and cognitive resources such as educational cookery classes, then the combined interventions are more likely to lead to behaviour maintenance. In addition, the map suggests that with sufficient self-regulation, behaviours are more likely to become habitual, which increases the likelihood of behaviour maintenance. One modelling approach would therefore be to assume the behaviour becomes habitual and is maintained within a percentage of the population, whilst it is not maintained within the remainder. This parameter could be elicited (in the absence of data) and then tested within sensitivity analysis.

When combining interventions within the model, based upon the behavioural map, it may be that the combination of some of the interventions would increase the probability of maintenance compared with only one intervention. For example, cookery classes and dietary advice which affects maintenance motivation and cognitive resources, in combination with the introduction of a new supermarket in a deprived area is more likely to lead to behaviour maintenance than either of the interventions alone. These probabilities could be elicited from behavioural scientists.

Figure 2: Illustrative behavioural map for dietary behaviours using behaviour maintenance themes from Kwasnicka et al. [11]


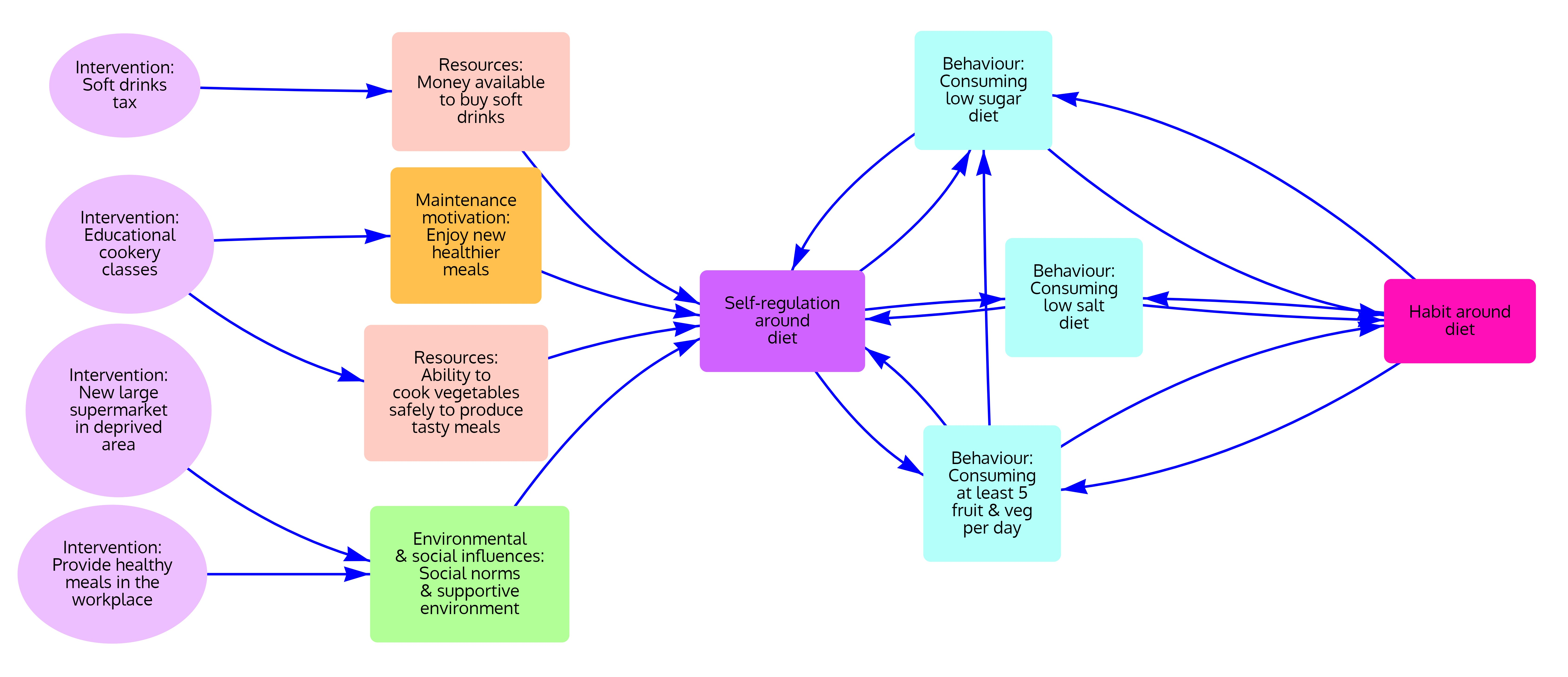


***5) Agent based modelling***

Obesity has been shown to spread within social networks [12]. The diet of a parent in a family will likely impact the diet of the other household members. Similarly, friends may influence each other’s levels of physical activity. These were not considered within this diabetes prevention project. For each intervention, whether these social network impacts would have been captured within the intervention study is evaluated below.

Soft drinks tax: This was provided across the population, and hence any social network impacts would have been captured by the study.

Workplace health promotion: This was provided within a workplace setting. Any social network impacts upon others within the same workplace should have been captured within the study. The intervention changed the access to healthy foods at work, and since motivation or capability were not altered by the intervention, it is unlikely that eating behaviour would have changed within other environments. Therefore, it would be unreasonable to assume that there were additional social network impacts of the intervention.

New large supermarket in deprived area to increase access to fruit and vegetables: Only a small proportion (<7%) of the households in the area completed the survey for this intervention which means that any social network impacts between households would mostly not have been picked up. In addition, there would have been potential impacts upon other household members. Thus, the impacts of the intervention may have been underestimated by the current model.

Dietary advice in high-risk communities: It is likely that the impact of the cookery classes would not only affect the diet of the female being given the intervention, but also the remainder of her household, particularly if she is the main cook in the household. Thus, the impacts of the intervention may have been underestimated by the current model.

Diabetes prevention programme (DPP): Changes in behaviour from the DPP may have impacts upon the behaviour of other people within their social networks, not captured by the studies. Thus, the impacts of the intervention may have been underestimated by the current model.

It would be possible to modify the current diabetes prevention model to incorporate these social network impacts, rather than needing to build a new model. Within the diabetes prevention model there is a Master model R code, that calls other scripts, such that each script can be written separately and is run in a certain order when it is called. There is an initial population which is generated with individual characteristics, and these are updated annually for the entire population for as long as the model is run. It would be necessary to create a social network structure to enable one individual’s behaviour to influence others. It is possible to do this in R using the built-in functions ‘sna’ and ‘network’ or ‘igraph’. Alternatively, other packages which are compatible with R, such as NetLogo, could be utilised for generating the social network structures and then the existing model R scripts loaded in. A search for social network analysis and fruit and vegetable consumption and physical activity suggested that social network analysis relating to these behaviours do exist, but not in the relevant population [13, 14]. These suggest that there are social network impacts upon the behaviours. The decision about whether to incorporate these effects within the model would depend upon the constraints around the decision-making process.

Within the diabetes model, when interventions reported behavioural outcomes such as consumption of fruit and vegetables, these were linked to one of the clinical risk factors using published literature. For example, studies of how vitamin C levels are related to the risk factor HbA1c, and how consumption of fruit and vegetables is related to systolic blood pressure were utilised. Similar evidence could be utilised for the relationship between behaviour and clinical risk factors within the model.

***6) Differential equation modelling***

Since the decision makers were interested in outcomes by multiple sociodemographic characteristics, differential equation modelling would be more challenging than an individual level modelling approach.

***7) Social network analysis***

Since it has been identified that social networks are important in this context, if resources were to allow, social network analysis could be undertaken and incorporated within the ABM.

***8) Geographical information systems (GIS)***

An intervention of interest to decision makers was local transport policy, however it was not assessed since this model would not improve upon existing models of physical activity. If this was a key priority for decision makers and individuals had been shown within a study to be more likely to cycle to work if given access to cycle lanes, for example, then geographical information system analysis (eg. using OpenStreetMap) could be utilised to model cycle lanes to estimate the cost-effectiveness of alternative cycle lane routes upon different demographics of the population. Similarly to the incorporation of social network analysis, it is possible to do this in R using the built-in functions, or packages which are compatible with R, such as NetLogo, could be utilised for generating and visualising the GIS data [15].

***9) Discrete event simulation (DES) with resource constraints***

The behaviours of consuming low salt and sugar diets and at least 5 fruit and veg per day may be influenced by access to healthy foods, but physical resource constraints within the system are not considered to be a key component influencing behaviour.

***10) Theory-informed statistical and econometric analyses***

There are several dietary and physical activity behaviours which may interact and impact the same outcomes of interest. These behaviours are complements i.e. improving physical activity will also improve diet. Therefore, if interventions relating to diet were clearly value for money, then it would not be necessary to undertake econometric analyses to determine the impact of diet on physical activity. Otherwise, a suitable dataset would need to be identified which collected longitudinal data on both the dietary behaviours and physical activity. The best dataset for this purpose within the UK is the Whitehall II dataset, since it collects a wide range of health, socioeconomic and behavioural data and is longitudinal rather than cross-sectional. Econometric input would be required for any analyses. The soft drinks tax intervention was based upon an econometric analysis already reported within the intervention study [16].

Since the initial diabetes prevention project, Bates [17] was given access to individual level data from a trial of a weight-management intervention reporting psychological and behavioural outcomes at four time points. The authors were therefore able to undertake latent growth curve analysis to assess the relationship between the intervention, the psychological mechanisms (dietary restraint, habit strength, autonomous diet regulation) and BMI. Interestingly, the study had collected variables around habit and self-regulation which are associated with behaviour maintenance. In this case study, there was no predictive advantage of incorporating the psychological mechanisms into the health economic model, but it became possible to assess the impact of the intervention upon subgroups in terms of their psychological mechanisms which could inform intervention targeting. This analysis could also be used for pre-trial modelling for new interventions.

There was one before and after study of a new large supermarket in a deprived area to increase access to fruit and vegetables; however, there was only one data point collected at each stage. It would therefore not be possible to undertake interrupted time series regression analysis given the data available for the interventions.

Within the diabetes model, latent growth curve modelling was used to account for changes to risk factors such as blood pressure and cholesterol at the individual level, and their impact upon changes to BMI at the individual level over time; however it did not account for population level increases in these risk factors. It is, however, known that population level BMI and other related risk factors have increased in the population over time. The reasons for this are complex but are in part due to increasingly sedentary jobs and easier access to unhealthy food and drink. Within other models, such as the UK Foresight Obesity model, the population-level changes in BMI have been modelled using regression analyses based upon repeated cross-sectional data from the Health Survey for England. However, this does not disentangle the effects of age, period and cohort, which are all likely to impact on diet and physical activity behaviours. Thus, undertaking this statistical analysis using data from the Health Survey for England would allow better modelling of these influences upon behaviour over time.

***11) Expert elicitation***

Within the modelling project, some of the modelling assumptions were informally elicited from clinical experts, but they focused upon clinical rather than behavioural outcomes and no formal methods were utilised. Behavioural scientists and clinical experts could have been invited to formally elicit parameters for the extrapolation of intervention effectiveness. This would, however, have required substantial additional resources, and so it would be necessary to weigh up the costs of doing this compared to informal elicitation from behavioural scientists throughout the project.

***12) Qualitative research/ process tracing***

In order to inform the causal relationships between the interventions and BMI, qualitative research could be undertaken to understand how and why the interventions might affect (or not affect) physical activity or diet over time. This could be done using process tracing, where behavioural theory is used to develop theory of change models which are then tested using qualitative data collection and analysis.

1. **The agent-based model of alcohol consumption**

The alcohol model [18-20] is an agent-based model of a US population which quantifies behavioural theory using individual-level data with the aim of being able to replicate population-level drinking patterns. It has successfully replicated 15 years of alcohol consumption patterns. Based upon the modelling work, it is possible to suggest the types of interventions which may be effective in reducing alcohol consumption. Within the currently published literature, the model does not include social networks. However, this is work in progress.

***What are the benefits of adapting this model into a health economic model?***

A health economic model aims to compare all the costs and benefits associated with the intervention(s) and the current system in a transparent way. This can then be used to inform policy decisions about which interventions to fund. Currently, the model is not able to inform policy decisions because it simulates only the current system. There is therefore a benefit to extending this model in order to inform policy.

The benefit to utilising an existing agent-based model if possible, rather than developing a new model is enabling the analysis to feasibly be undertaken within a decision making process. The current model has been developed over several years, and this would not be practical for informing policy if each model were to begin de novo.

***What is needed for the current simulation to be developed into a health economic model?***

In order to develop the existing ABM into a health economic model:

(a) alcohol consumption would need to be predicted over the lifetime of the population, both with and without the interventions of interest;

(b) a relationship between alcohol consumption and a set of related diseases would need to be defined so that the difference in prevalence of each of the diseases could be estimated; and

(c) costs and utilities would need to be applied to each of the diseases.

(b) and (c) above have been done previously within another health economic model [21]. Published data was used to relate alcohol consumption to relative risks of hospital admission and mortality for a set of 47 chronic and acute alcohol-related conditions. Costs were sourced and estimated for hospital admissions and each of the conditions and utilities from published sources were applied to each of the conditions, adjusted for age and sex. It would be possible to update these estimates and incorporate them into the ABM.

For (a), practically it would be relatively simple to extend the time horizon of the model. There is uncertainty about the validity of using the behavioural theories for prediction over a much longer time period than the data provided; however, it could be argued that this is a better approach than extrapolating current data without the use of behavioural theory. It would, therefore, be important to set out the uncertainties associated with the approach.

If individual level data existed for the variables from the behavioural theories for the intervention(s), then it would be possible to use the same approach as for modelling the current system. Since this is unlikely, then it would be necessary to make some assumptions about how the intervention(s) would impact the variables over time. This would require use of the taxonomy, in particular: (1) collaboration with behavioural scientists, (2) behavioural mapping, (3) identifying upon which theories interventions were developed, if reported, and undertaking literature searches for maintenance studies, as well as (4) applying the behaviour change techniques taxonomy from the behaviour change wheel framework to describe interventions to model. This could be done using the same process as for the diabetes prevention example.

**References**

1. Breeze PR, Thomas C, Squires H, Brennan A, Greaves C, Diggle P, et al. Cost-effectiveness of population-based, community, workplace and individual policies for diabetes prevention in the UK. Diabet Med. 2017;34(8):1136-44. doi: 10.1111/dme.13349.

2. Dansinger ML, Tatsioni A, Wong JB, Chung M, Balk EM. Meta-analysis: the effect of dietary counseling for weight loss. Ann Intern Med. 2007;147(1):41-50. doi: 10.7326/0003-4819-147-1-200707030-00007.

3. Chater AM, Smith L, Ferrandino L, Wyld K, Bailey DP. Health behaviour change considerations for weight loss and type 2 diabetes: nutrition, physical activity and sedentary behaviour. Practical Diabetes. 2020;37(6):228-31b.

4. Murray JM, Brennan SF, French DP, Patterson CC, Kee F, Hunter RF. Mediators of Behavior Change Maintenance in Physical Activity Interventions for Young and Middle-Aged Adults: A Systematic Review. Ann Behav Med. 2018;52(6):513-29. doi: 10.1093/abm/kay012.

5. Fjeldsoe B, Neuhaus M, Winkler E, Eakin E. Systematic review of maintenance of behavior change following physical activity and dietary interventions. Health Psychol. 2011;30(1):99-109. doi: 10.1037/a0021974.

6. Howlett N, Trivedi D, Troop NA, Chater AM. Are physical activity interventions for healthy inactive adults effective in promoting behavior change and maintenance, and which behavior change techniques are effective? A systematic review and meta-analysis. Transl Behav Med. 2019;9(1):147-57. doi: 10.1093/tbm/iby010.

7. Madigan CD, Fong M, Howick J, Kettle V, Rouse P, Hamilton L, et al. Effectiveness of interventions to maintain physical activity behavior (device-measured): Systematic review and meta-analysis of randomized controlled trials. Obesity Reviews. 2021;22(10). doi: 10.1111/obr.13304.

8. Murray JM, Brennan SF, French DP, Patterson CC, Kee F, Hunter RF. Effectiveness of physical activity interventions in achieving behaviour change maintenance in young and middle aged adults: A systematic review and meta-analysis. Social Science & Medicine. 2017;192:125-33. doi: 10.1016/j.socscimed.2017.09.021.

9. Michie S, West R, Finnerty AN, Norris E, Wright AJ, Marques MM, et al. Representation of behaviour change interventions and their evaluation: Development of the Upper Level of the Behaviour Change Intervention Ontology. Wellcome Open Res. 2020;5:123. doi: 10.12688/wellcomeopenres.15902.2.

10. Gilbert N: Participatory System Mapper (PRSM). Accessed Sept 2023.

11. Kwasnicka D, Dombowski SW, M., Sniehotta F. Theoretical explanations for maintenance of behaviour change: a systematic review of behavioural theories. Health Psychol Rev. 2016;10(3):277-96.

12. Christakis NA, Fowler JH. The Spread of Obesity in a Large Social Network over 32 Years. N Engl J Med. 2007;357:370-9.

13. Choi YJ, Ailshire JA, Crimmins EM. Living alone, social networks in neighbourhoods, and daily fruit and vegetable consumption among middle-aged and older adults in the USA. Public Health Nutr. 2020;23(18):3315-23. doi: 10.1017/S1368980020002475.

14. Storey KE, Stearns JA, McLeod N, Montemurro G. A social network analysis of interactions about physical activity and nutrition among APPLE schools staff. SSM Popul Health. 2021;14:100763. doi: 10.1016/j.ssmph.2021.100763.

15. Crooks A, Malleson N, Manley E, Heppenstall A. Agent-based modelling & geographical information systems. A practical primer. London: SAGE Publications Ltd.; 2019.

16. Briggs AD, Mytton OT, Kehlbacher A, Tiffin R, Rayner M, Scarborough P. Overall and income specific effect on prevalence of overweight and obesity of 20% sugar sweetened drink tax in UK: econometric and comparative risk assessment modelling study. BMJ. 2013;347:f6189. doi: 10.1136/bmj.f6189.

17. Bates S. Incorporating Psychological Mechanisms of Action in a Health Economic Model of Obesity. 2021.

18. Buckley C, Field M, Vu TM, Brennan A, Greenfield TK, Meier PS, et al. An integrated dual process simulation model of alcohol use behaviours in individuals, with application to US population-level consumption, 1984-2012. Addictive behaviors. 2022;124:107094-. doi: 10.1016/j.addbeh.2021.107094.

19. Vu TM, Probst, C., Nielsen, A., Bai, H., Buckley, C., Meier, P. Strong, M., Brennan, A., Purshouse, R. A Software Architecture for Mechanism-Based Social Systems Modelling in Agent-Based Simulation Models. Journal of artificial societies and social simulation : JASSS. 2020;23(3):1.

20. Probst C, Vu TM, Epstein J, Nielsen E, Buckley C, Brennan A, et al. The normative underpinnings of population-level alcohol use: an individual-level simulation model. Health Educ Behav. 2020;47(2):224-34.

21. Purshouse RC, Meier PS, Brennan A, Taylor KB, Rafia R. Estimated effect of alcohol pricing policies on health and health economic outcomes in England: an epidemiological model. Lancet. 2010;375(9723):1355-64. doi: 10.1016/S0140-6736(10)60058-X.
